# Metformin Inhibits Growth of Human Glioblastoma Cells and Enhances Therapeutic Response

**DOI:** 10.1371/journal.pone.0123721

**Published:** 2015-04-13

**Authors:** Julie Sesen, Perrine Dahan, Sarah J. Scotland, Estelle Saland, Van-Thi Dang, Anthony Lemarié, Betty M. Tyler, Henry Brem, Christine Toulas, Elizabeth Cohen-Jonathan Moyal, Jean-Emmanuel Sarry, Nicolas Skuli

**Affiliations:** 1 INSERM U1037, Centre de Recherche en Cancérologie de Toulouse, Toulouse, France; 2 Department of Neurosurgery, Johns Hopkins University, Baltimore, Maryland, United States of America; University Hospital of Navarra, SPAIN

## Abstract

High-grade gliomas, glioblastomas (GB), are refractory to conventional treatment combining surgery, chemotherapy, mainly temozolomide, and radiotherapy. This highlights an urgent need to develop novel therapies and increase the efficacy of radio/chemotherapy for these very aggressive and malignant brain tumors. Recently, tumor metabolism became an interesting potential therapeutic target in various cancers. Accordingly, combining drugs targeting cell metabolism with appropriate chemotherapeutic agents or radiotherapy has become attractive. In light of these perspectives, we were particularly interested in the anti-cancer properties of a biguanide molecule used for type 2 diabetes treatment, metformin. In our present work, we demonstrate that metformin decreases mitochondrial-dependent ATP production and oxygen consumption and increases lactate and glycolytic ATP production. We show that metformin induces decreased proliferation, cell cycle arrest, autophagy, apoptosis and cell death *in vitro* with a concomitant activation of AMPK, Redd1 and inhibition of the mTOR pathway. Cell sensitivity to metformin also depends on the genetic and mutational backgrounds of the different GB cells used in this study, particularly their PTEN status. Interestingly, knockdown of AMPK and Redd1 with siRNA partially, but incompletely, abrogates the induction of apoptosis by metformin suggesting both AMPK/Redd1-dependent and –independent effects. However, the primary determinant of the effect of metformin on cell growth is the genetic and mutational backgrounds of the glioma cells. We further demonstrate that metformin treatment in combination with temozolomide and/or irradiation induces a synergistic anti-tumoral response in glioma cell lines. Xenografts performed in nude mice demonstrate *in vivo* that metformin delays tumor growth. As current treatments for GB commonly fail to cure, the need for more effective therapeutic options is overwhelming. Based on these results, metformin could represent a potential enhancer of the cytotoxic effects of temozolomide and/or radiotherapy.

## Introduction

In accordance with the World Health Organization (WHO) classification, Glioblastoma (GB) are grade IV astrocytic brain tumors [1, 2]. The American Association of Neurological Surgeons estimates that 15% of diagnosed brain tumors are glioblastomas and therefore the most common brain tumors in adults. GB are one of the most aggressive and deadly human cancers and despite surgery, radiotherapy and chemotherapy, the median survival is approximately 12–14 months. The standard treatment is surgery, if possible, followed by the combination of temozolomide (TMZ) and radiotherapy [3]. Both ionizing radiations and temozolomide induce DNA damage, which can lead to cell death [4, 5]. TMZ particularly exerts its cytotoxic effects by methylating the guanine bases in DNA to form O-6-methylguanine and this induces mispairing and consequent defects in DNA replication. Repair of this damage by the O6-methylguanine methyltransferase (MGMT) is associated with resistance to TMZ and methylation of the MGMT promoter, thus allowing predictability of drug sensitivity for GB patients [6]. Recent data indicate that TMZ-induced apoptosis involves the activation of the energy-sensing kinase [7], adenosine monophosphate-activated protein kinase (AMPK), and suggest that increased AMPK activity could enhance GB cytotoxicity mediated by TMZ and radiation. Interestingly, recent studies have also demonstrated that targeting metabolic pathways may be an effective therapeutic strategy in several types of cancer [8, 9].

Metformin, a member of the biguanide family, is the most commonly used oral normoglycemic agent for type 2 diabetes [10] and exhibits anti-tumoral effects. To date, two major effects of metformin have been described: inhibition of mitochondrial electron transport chain complex I (ETCI) and Liver Kinase B1 (LKB1)-dependent and independent activation of AMPK, a regulator of energy homeostasis, metabolism and protein synthesis through inhibition of mammalian target of rapamycin (mTOR) [11, 12]. By targeting ETCI, metformin mediates changes in AMP/ATP ratios, calcium levels and mitochondrial transmembrane potential, which correlate with increased oxidative stress [13]. These effects lead to a local and whole-body increase in catabolism and mitochondrial biogenesis while inhibiting protein synthesis and anabolic pathways. *In vitro* and *in vivo* inhibition of cell growth is observed in leukemia, pancreatic, colon, prostate, ovarian, breast cancer, melanoma, endometrial, lung, glioma and hepatocellular carcinoma cells in response to metformin [14, 15]. The mechanistic role of AMPK in metformin’s anti-cancer activity, however, is still under debate in most cancers, including gliomas. On one hand, some studies describe that glioma cells showed AMPK-dependent effects, such as decreased proliferation, a block in G_0_/G_1_ cell cycle progression and induction of cell death in U87 cell line, that were supported by an AMPK inhibitor, Compound C or AICAR. [16–18]. Additionally, metformin may target tumor-initiating stem cell-like glioma cells, also called Glioma Stem Cells (GSC), through AMPK-dependent inhibition of FOXO3 and AKT [18]. On the other hand, a recent study showed that AMPK was constitutively active in gliomas and that the anti-proliferative effects of metformin on GSCs were AMPK-independent [19]. Thus, the understanding of metformin’s molecular response should be improved and the therapeutic opportunity of its use in combination with current treatments should assess the clinical relevance, as these treatments commonly fail to cure gliomas.

Our work summarizes experimental evidence that metformin has potential to enhance the cytotoxic effects of TMZ and radiotherapy. First, we demonstrate that metformin decreases mitochondrial oxygen consumption and increases lactate and glycolytic ATP production in four GBM cell lines. Interestingly, our work shows that metformin induces decreased proliferation, cell cycle arrest, autophagy, and apoptotic cell death *in vitro* in part *via* AMPK and Redd1 activation and inhibition of mTOR pathways, but differentially affects glioma cell growth depending on the genetic and mutational backgrounds, particularly their PTEN status. These *in vitro* effects led *in vivo* to a marked delay of tumor growth. Finally, we demonstrate that metformin treatment in combination with TMZ or irradiation induces a synergistic anti-tumoral response in glioma cell lines through increased cell death. Based on these results, metformin might significantly potentiate the cytotoxic effects of TMZ and/or radiotherapy.

## Materials and Methods

### Cell lines and culture conditions

Four human glioblastoma cell lines, U87 (ATCC HTB-14), LN18 (ATCC CRL-2610), U251 and SF767 (obtained from M. Celeste Simon’s laboratory, University of Pennsylvania, Philadelphia, PA, USA), were used and routinely maintained in Dulbecco’s Modified Eagle Medium (DMEM, Lonza) supplemented with 10% fetal calf serum (Lonza) at 37C° in 5% CO_2_-humidified incubators and were subcultured once or twice a week as described previously [20]. Cells were treated or not with the following compounds, 1-10mM metformin (Sigma-Aldrich), 5–100μM temozolomide (Schering-Plough Europe), 10μM bafilomycin (Sigma-Aldrich) and irradiated or not at 5Gy using the Gammacell 40 Exactor irradiator (Nordion).

### Oxygen consumption rate (Seahorse XF24 assay)

Oxygene consumption rate (OCR) was measured in real time by Seahorse XF24 (Seahorse Bioscience) [21]. The day before the assay, metformin-treated and untreated cells were plated in 24-well cell culture microplates (Seahorse Bioscience) at 70,000 cells per well in quadruplicate by conditions. Prior to this XF assay cells were incubated at 37°C for 60min in assay medium (XF Base Medium, Seahorse Bioscience) supplemented by Glutamine 2mM (Life technologies), Glucose 10mM (Sigma-Aldrich) and Sodium Pyruvate 2mM (Sigma-Aldrich) as finals concentrations and adjusted to pH 7.4, as recommended by manufacturer. Normalization was performed by determining the protein content in each well. Briefly, cells were lysed after XF assay by NaOH 0.1M for 45min before running a standardized protein assay in order to express the OCR results in pmol/min/μg of proteins.

### ATP measurement

ATP assays were performed with Cell Titter-Glo 2.0 assay (Promega) after appropriate treatment. Ninety six-well plates were seeded with 10,000 cells per well in 80μL of cell culture media (Lonza). 20μL of inhibitors, Oligomycine (Sigma-Aldrich), FCCP (Sigma-Aldrich) and Sodium iodoacetate (Sigma-Aldrich) were added at final concentration of 1.3μM, 30μM and 100μM respectively. After 1hr at 37°C CO_2_ 0.5%, 100μL of Cell Titer-Glo were added to each well to a final volume of 200μL. After 10min at RT with and then without agitation, the plate was read by luminometer (FluoStar, Optima). By comparing the different conditions, global ATP and percentages of both glycolytic and mitochondrial ATP were determined [22].

### Lactate measurement

This assay was performed using Lactate Colorimetric Assay kit (Biovision) according to manufacturer’s instructions. Cell culture supernatants, treated or not, pre-diluted 1:3 in cell culture media (Lonza) were used with three replicates per condition. A standardized protein assay based on cell density was performed to normalize the results.

### Mitochondria isolation and Complex I activity measurement

Mitochondria were isolated from cell treated with or without Metformin 10mM for 48hrs as previously described [23]. Briefly, cells were lysed on ice with lysis buffer (20mM HEPES pH 7.4, 1mM EGTA, 1mM EDTA, 1.5mM MgCl_2_, 10mM KCL, 0.25mM sucrose, Protease Inhibitor (Sigma-Aldrich)) and homogenized 15 times with a dounce homogenizer. Then, cells were centrifuged twice at 3000rpm for 10min at 4°C in order to completely remove cell debris and nuclei. Finally, the supernatant was subjected to centrifugation at 13,000rpm (20min, 4°C) to separate mitochondrial fractions from the cytoplasm. Mitochondrial pellets were resuspended in lysis buffer and kept at −80°C. The rotenone-sensitive activity of respiratory electron transport chain (ETC) complex I was measured spectrophotometrically, as described previously [24], by measuring the ubiquinone-dependent reduction of DCPIP (Dichlorophenolindophenol). Briefly, 10μg of mitochondria were resuspended in 200μL of phosphate buffer (35mM, pH 7.3) supplemented with 2mM KCN, 2μg/mL AntimycinA (Sigma-Aldrich), 5mM MgCl2, 130μM NADH, 60μM CoQ (Sigma-Aldrich) and 88μM DCPIP. The absorbance at 600nm was recorded every 15sec during 3min (FLUOstar Optima, BMG Labtech), monitoring the extinction of DCPIP at 37°C (*ε* = 21mM^−1^ cm^−1^). Results were expressed as relative activities compared to untreated cells. All the reagents and chemicals are from Acros Organics (Fisher), unless stated.

### Proliferation assay

GBM cells (2.5x10^4^) were treated or not with 10mM metformin and/or 5–50μM temozolomide or Phosphate Buffered Saline (PBS, Lonza) as vehicle. Cells were then collected at the indicated time in 1mL of trypsin (Lonza) and washed in PBS. 20μL of cell suspension were mixed with 20μL of Trypan Blue (Lonza). Cells were counted using Malassez slide (Invitrogen, Life Technologies) and the number of cells per milliliter was determined by the following formula: (Cell number/20 squares)x2x100x1000. Pictures were also taken with a Nikon microscope (NIS Element, Nikon) during the course of the assay.

### Flow Cytometry

Forty-eight hours after treatment with metformin (10mM), temozolomide (100μM) and/or ionizing radiations (5Gy), cells were collected, washed in PBS and prepared for flow cytometry. For cell death, we used reagents and protocols from the FITC Annexin V/Dead Cell Apoptosis Kit (Invitrogen Life Technologies). Briefly, we incubated cells for 15min at RT in 100μL of Annexin-binding buffer 5X (10mM HEPES pH7.4, 140mM NaCl, 2.5mM CaCl_2_), containing 5μL of Annexin V-FITC antibody and 1μL of Propidium Iodide (PI) solution at 100μg/mL. 400μL of Annexin-binding buffer 5X were then added after washes with PBS-BSA 1%. For cell cycle analysis, cells were fixed with cold ethanol 100% and then permeabilized with Triton X-100 at 0.25%. Cells were then labeled with FITC-anti-Ki67 antibody (Abcam) during 45min at RT and treated with RNAse A at 1μg/mL before labeling with PI during 2hrs at RT. For autophagy analysis, cells were labeled with Acridine Orange (1:10000 dilution, Fluka) for 20min at RT and washed with PBS. Labeled cells were preserved on ice and run on a flow cytometer (FACS Calibur, Becton-Dickinson).

### Western Blot analysis

Cells treated or not with metformin (10mM), were lysed in 70μL of lysis buffer (50mM Tris HCl pH 7.5, 0.1% Triton, 5mM EDTA complemented by protease (Chemicon Millipore) and phosphatase (Sigma-Aldrich) inhibitors. Western blots were performed as previously described using monoclonal rabbit antibodies [20, 22], anti-LC3b (1/1000, Cell Signaling), anti-Beclin 1 (1/1000, Cell Signaling), anti-p62 (1/1000 Abcam), anti-phospho (T172) AMPK (1/1000, Cell Signaling), anti-AMPK (1/1000, Cell Signaling) anti-phospho (S79) ACC (1/1000, Cell Signaling), anti-ACC (1/1000, Cell Signaling), anti-phospho (S2448) mTOR (1/1000, Cell Signaling), anti-mTOR (1/1000, Cell Signaling), anti-phospho (T389) p70S6 Kinase (1/1000, Cell Signaling), anti-p70S6 Kinase (1/1000, Cell Signaling), anti-phospho (T37/46) 4EBP1 (1/1000, Cell Signaling), anti-phospho (S473) AKT (1/1000, Cell Signaling), anti-phospho (T308) AKT (1/1000, Cell Signaling), anti-AKT (1/1000, Cell Signaling), anti-HIF-1α (1/1000, Cayman Chemical), anti-Redd1/DDIT4 (1/1000, Abcam and Proteintech) and were normalized using a rabbit polyclonal antibody anti-β-tubulin (1/1000, Cell Signaling). Gel quantification was performed using ImageJ (Windows 1.47, Research Services Branch, NIH).

### siRNA Transfection

Human glioblastoma cells (2x10^5^) were transfected with either 500nM or 50nM of siControl (siCtrl, Qiagen) or 500nM of specific siRNA for AMPKα1 and α2 (siAmpk, InVitrogen) or 50nM of a pool of specific siRNA for Redd1/DDIT4 (ON-TARGET plus SMART pool siRNA, Dharmacon) before metformin treatment and as described previously [22]. Small interfering RNA transfection was performed using Lipofectamine RNAimax reagent (Invitrogen Life Technologies) following manufacturer’s instructions.

### Xenograft experiments

Xenograft tumors were generated by injecting either U87 (5x10^5^ cells) or LN18 (1x10^6^ cells) human glioblastoma cells, in 100μL of PBS, subcutaneously on both flanks of NU/NU athymic mice (n = 5 mice (10 tumors) per group, Charles River). After tumor formation, mice were given daily intraperitoneal injections with 200μL of 300mg/kg/day metformin or vehicle (PBS). Tumor dimensions were measured with a caliper on days indicated and volume calculated using the formula: v = π/6xAxBxB, where A is the larger diameter and B is the smaller diameter. At the end of the experiment, mice were anesthetized with an intraperitoneal injection of a ketamine (100mg/kg) / xylazine (100mg/kg) mix and sacrificed by cervical dislocation. Then, tumors were dissected, weighed, photographed and fixed with alcohol formalin acetic acid fixative for 48hrs. Tumors were embedded in paraffin and sectioned for immunohistochemistry. All procedures were performed in accordance with the guidelines set forth by INSERM and approved by the Comités d'Ethique en Expérimentation Animale du Ministère de l'Enseignement Supérieur et de la Recherche.

### Immunochemistry

Proliferation, cell death and autophagic processes in xenografts were determined by immunohistochemistry for Ki67 marker using an anti-Ki67 antibody (Sigma-Aldrich), for active caspase-3 marker using an anti-active caspase-3 antibody (R&D Systems) and for LC3b-II marker using an anti-LC3b-II antibody (Cell Signaling), respectively. Hematoxylin and Eosin (H&E) staining was also performed. For quantification, Ki67 positively stained cells in six consecutive and independent fields were counted from the edge towards the center of each section. Photographs for quantification were taken with a Leica DM4000B microscope (Leica).

### Statistical analysis

Unpaired t-test was used to calculate final P-values. Data are representative of at least three independent experiments and significance is represented by * or # in which */#p<0.05, **/##p<0.01, ***p<0.001.

## Results

### Metformin impairs mitochondrial functions and favors glycolysis in human glioma cells

**Fig 1:**
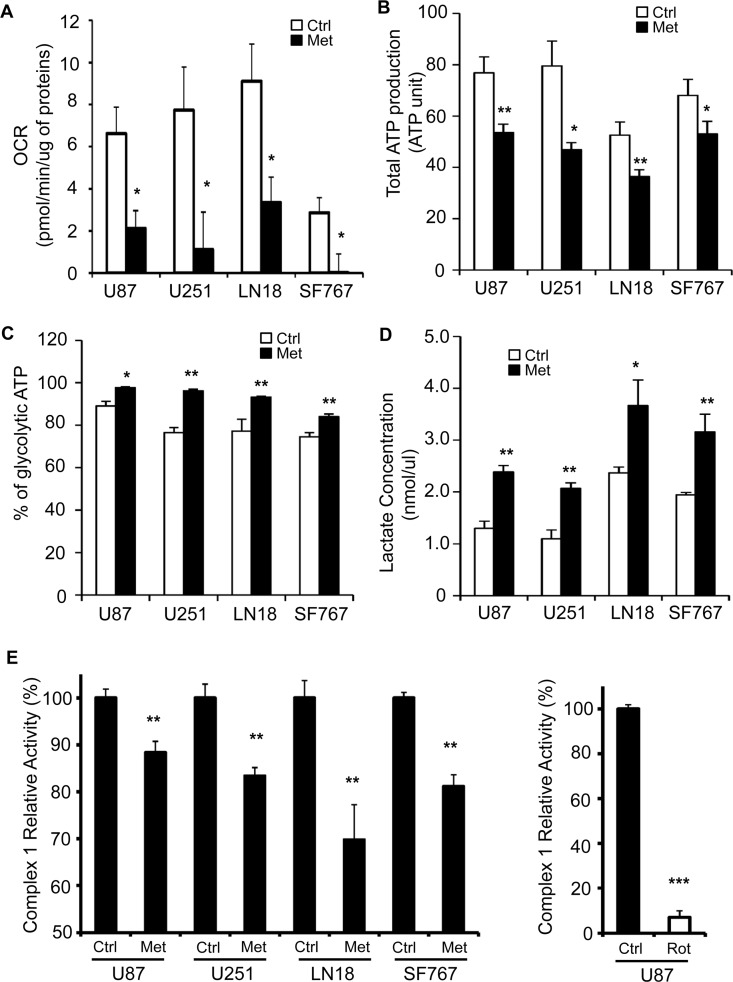
Metformin inhibits mitochondria and induces a shift towards glycolytic metabolism in human glioblastoma cells. (**A**) Four human GB cell lines (U87, U251, LN18 and SF767) were treated with or without metformin (10mM) for 48hrs and Oxygen Consumption Rate (OCR, pmol/min) was measured using Seahorse XF24 Mito stress assay. Data were normalized to total protein content of each well. Metformin treatment significantly reduces OCR (*p<0.05 vs. Control, n = 4). (**B**) Total ATP production and (**C**) glycolytic ATP were determined in metformin-treated or not U87, U251, LN18 and SF767 GB cell lines (*p<0.05, **p<0.01 Met vs. Ctrl, n = 3). (**D**) Lactate concentration (nmol/μL) in U87, U251, LN18 and SF767 cell culture supernatants was determined and shows increased lactate production in response to metformin treatment (*p<0.05, **p<0.01 Met vs. Ctrl, n = 3). (E) Effects of Metformin on rotenone-sensitive ETCI complex activity. (Left panel) GB Cells were treated or not for 48hrs with metformin (Met, 10mM). Mitochondria were isolated and complex I activity was assessed. (Right panel) 10μg of isolated mitochondria from untreated U87 cells were treated for 1hr by rotenone (Rot, 10μM) immediately before the complex I enzymatic assay. Results, expressed as mean ± SEM of at least three independent experiments, represent Complex I relative activities compared to the related control. (**p<0.01, ***p<0.001 compared with the related control).

Human glioma cells and particularly glioma stem cells (GSC) have previously been shown to be sensitive to metformin [19, 25, 26], which is known to inhibit ETCI. First, we aimed to assess respiratory characteristics and examined whether or not metformin could inhibit cellular oxygen consumption in these cells. We specifically selected four different human glioma cell lines, U87, U251, LN18 and SF767 to have a panel of cells with different mutations (p53, PTEN, and MGMT), and sensitivity to temozolomide treatment generally found in glioblastoma (S1A Fig). We treated these glioma cells with 10mM of metformin for 48hrs and subsequently determined oxygen consumption rate. Not surprisingly, metformin decreased oxygen consumption by a 3- to 4-fold in all treated cells, regardless their respective basal respiratory rate (Fig 1A). Then, we determined whether this decreased oxygen consumption could lead to diminished ATP production and glycolysis stimulation as so called Pasteur Effect [27]. These measurements revealed that total ATP production was significantly decreased in all metformin-treated GB cell lines (Fig 1B) and we also observed that the decrease in global ATP production was accompanied by an increase in glycolytic ATP production (Fig 1C). Of note, the tested GB cell lines appear to already rely highly on glycolysis for ATP generation in the basal status as nearly 70–80% of their ATP comes from glycolysis, reaching almost 90–95% when cells are treated with metformin (Fig 1C). As other surrogates of the Pasteur Effect [27], we also measured lactate production and release to the extracellular medium 48hrs after metformin treatment. In correlation with the previous data, lactate concentration in the media of metformin-treated cells was increased by 2-fold (Fig 1D), suggesting that metformin treatment favors glycolysis in these GB cells. Finally, to more accurately determine how metformin could affect oxygen consumption as well as ATP/lactate production, we directly measured mitochondrial electron transport chain complex 1 activity (ETCI) after metformin treatment in our GB cell lines (Fig 1E). As shown in Fig 1E, ETCI activity is mildly but highly significantly decreased by 12% and up to 31% compared to control, in response to metformin treatment in all GB cell lines. Of note, we used rotenone, another specific mitochondrial complex 1 inhibitor, as a control for our assay, and we observed a much stronger inhibition of ETCI activity suggesting that metformin is not as potent of an ETCI inhibitor as rotenone and may have additional targets, other than ETCI, of which inhibition could lead to the global effect we see on GB cell mitochondria (Fig 1E). Altogether, these results demonstrate that metformin affects mitochondria functions leading to an increase of the glycolytic pathway towards lactate production in human GB cells.

### Metformin inhibits glioma cell proliferation through cell cycle arrest and cell death

**Fig 2:**
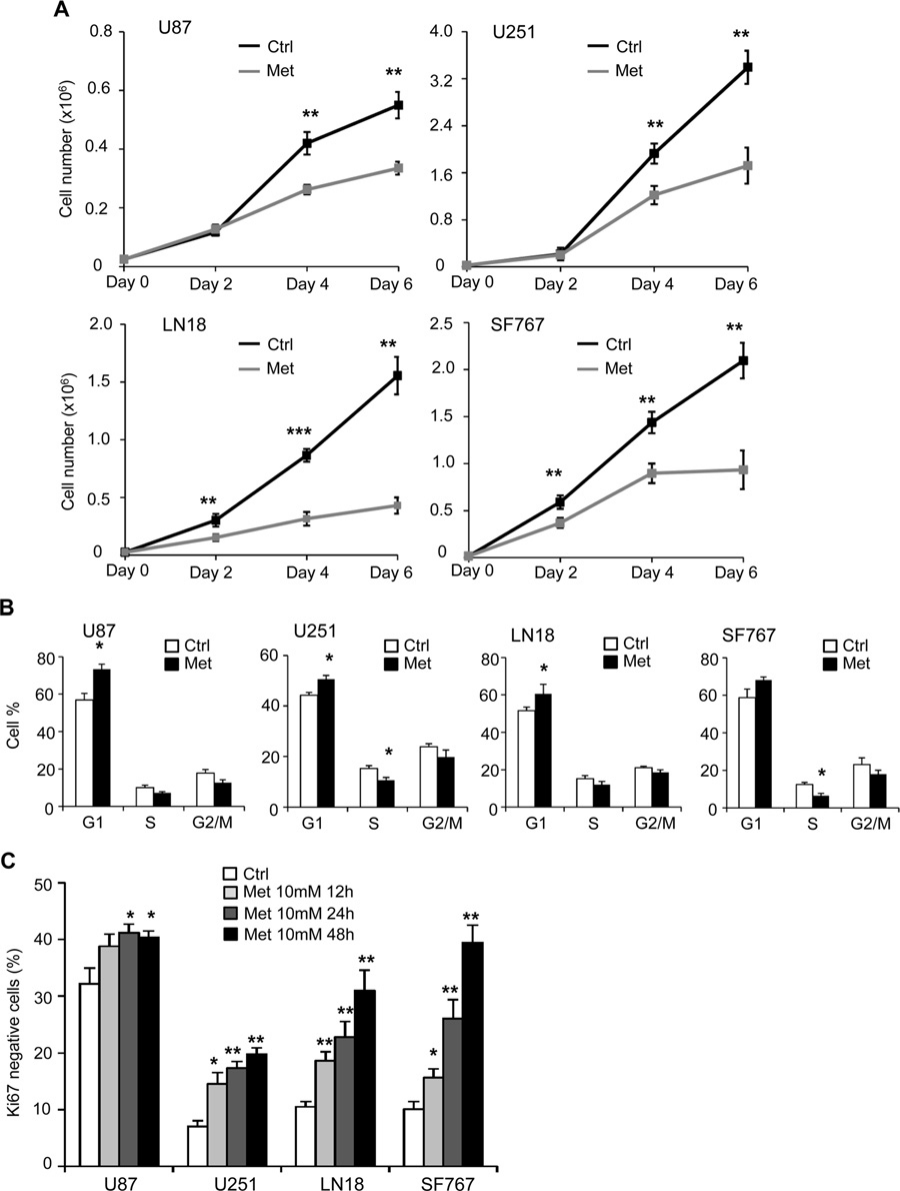
Metformin inhibits glioma cell proliferation and induces a transition in G_0_. (**A**) Proliferation assays performed with U87, U251, LN18 and SF767 cells show a decreased cell number in presence of metformin (black curve, Ctrl: PBS vehicle control; grey curve, Met: metformin 10mM) (*p<0.05, **p<0.01, ***p<0.001 Met vs. Ctrl, n = 3). (**B**) Quantification of cell cycle distribution of GB cells, using flow cytometry and PI, staining 48hrs after treatment. Metformin (Met) induces decreased cell number in S phase and increased cell number in G_1_ phase compared to control cells (Ctrl). (**C**) Quantification of GB cells in G_0_ phase, using flow cytometry and Ki67/PI staining, 12hrs, 24hrs and 48hrs after treatment. Metformin significantly increases the number of cells in G_0_ phase (*p<0.05, **p<0.01 Met compared to Ctrl, n = 3).

It is known that metformin affects viability and proliferation of several cancer cell lines. In order to investigate the effect of metformin on glioma cells, we treated different human GB cells (U87, U251, LN18 and SF767) with 1mM, 5mM and 10mM of metformin, and assessed viable cell number over 6 days (Fig 2A, S1B and S1C Fig and data not shown). As shown in Fig 2A, metformin treatment (10mM) leads to a significant decrease in all GB cell proliferation. LN18 and SF767 cells (PTEN WT) show an increased sensitivity to metformin with an anti-proliferative effect starting at 48hrs after treatment compared to U87 and U251 cells (PTEN mutated), where the effect is observed particularly at 96hrs. To assess the direct effect of metformin on GB cell proliferation and exclude toxicity from indirect effects of metformin-induced changes of cell metabolism such as glucose exhaustion or acidification of the extracellular media, we repeated this time-course experiment and supplied GB cell with fresh media every day for the time of the assay (S1C Fig). Interestingly, in these conditions, the metformin effect on cell proliferation is observed at later time points, four-five days for LN18, SF767 and U251 cells, and six days for U87 cells, suggesting that indirect metformin toxicity could be in part responsible for the decrease in GB cell number (S1C Fig). Of note, LN18 and SF767 cells (PTEN WT) were again more sensitive to metformin treatment compared to U87 and U251 cells (PTEN mutated) in these last conditions.

**Fig 3:**
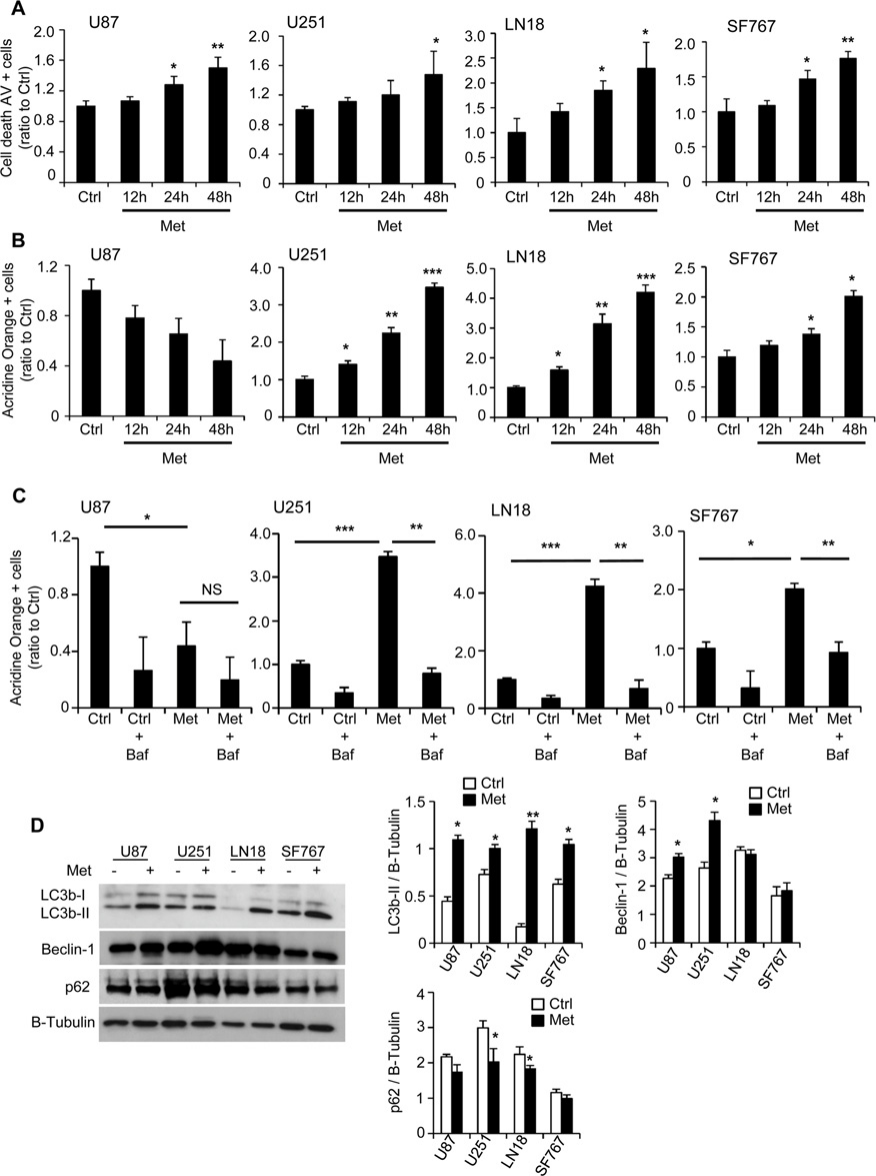
Metformin increases GB cell death and induces autophagic processes. (**A**) Quantification of apoptotic and necrotic cell death, using flow cytometry and Annexin-V/PI staining, 12hrs, 24hrs and 48hrs after treatment. Metformin significantly increases the number of AV positive cells (*p<0.05, **p<0.01 compared to control, n = 3). (**B-C**) Quantification of vesicle acidification, using flow cytometry and Acridine Orange staining, following 12hrs, 24hrs or 48hrs treatment in presence or not of 10mM metformin (B) and/or 10μM bafilomycin (C). Metformin increases vesicle acidification for U251, LN18 and SF767 cells. This effect can be reversed using the autophagy inhibitor, bafilomycin (NS: Non-Significant, *p<0.05, **p<0.01, *** p<0.001 n = 5). (**C**) Western Blot analyses for LC3b, Beclin-1 and p62 and respective quantifications showing induction of autophagy in response to metformin treatment (*p<0.05, **p < 0.01 compared to control, n = 3).

In order to explain the decreased proliferation with metformin treatment, we first looked at cell cycle in our human GB cells. We demonstrate that metformin treatment leads to a modest but significant block in the G_1_ phase for U87, U251 and LN18 cells compared to control (Ctrl) untreated cells and a similar tendancy in SF767 (Fig 2B). We also observed an increase in the number of cells in G_0_ (Ki67-negative cells) throughout the time course of the experiment in the presence of metformin (Fig 2C and S2A Fig). These results indicate that inhibition of GB cell growth by metformin treatment might be due to a G_1_ phase cell cycle arrest and transition into G_0_. Cell death and apoptosis often are consequences of the G_0_/G_1_ arrest. As such, the apoptotic cell death rate, reflected by the percentage of Annexin-V positive cells, was significantly increased in all GB cell lines in response to 48hr-metformin treatment compared to the control cells (Fig 3A and S2B Fig). Interestingly, the time course experiments allowed us to clarify the timeline of the different processes. More specifically, we were first able to detect cell cycle arrest as early as 12hrs after metformin treatment, reflected by the increased percentage of Ki67 negative cells (Fig 2C). Subsequent, cell death was consistently observed at 24hrs but mostly 48hrs following metformin treatment (Fig 3A). Taken together, these observations clearly indicate that metformin inhibits human glioma cell proliferation through the induction of cell cycle arrest, which would precede cell death processes.

### Metformin also induces autophagic process in human glioma cells

Metformin has been shown to induce autophagic processes in various cell types [28–31]. We analyzed the autophagic rate by flow cytometry, using acridine orange, and western blot on autophagy-related proteins such as LC3b-II, Beclin-1 and p62, respectively, in the four different GB cell lines. Two days following metformin treatment (10mM), acridine orange staining was significantly increased compared to control (Ctrl) cells (3.4-fold for U251, 4.2-fold for LN18 and 2-fold for SF767), except for U87 cell line, suggesting vacuole acidification within the GB cells (Fig 3B and S2C Fig). Indeed, time course experiment reveals that vacuole acidification begins at 12hrs but occurs primarily at 24hrs for the U251, LN18 and SF767 cells after metformin treatment (Fig 3B). In correlation with the data on cell cycle and cell death (Figs 2C and 3A), these results suggest that metformin affects GB cell cycle and would then induce autophagic and subsequent GB cell death processes. Furthermore, bafilomycin A1 treatment, a known inhibitor of the late phase of autophagy, led to a complete and significant reversal of glioma cell vesicle acidification for U251, LN18 and SF767 cells confirming the presence of autophagic flux induced in response to metformin (Fig 3C). In addition, we observed a significant increase in LC3b-II expression in all cell lines and an increase in Beclin-1 level in U87 and U251 cell lines 48hrs after metformin treatment (Fig 3D). At the same time, U251 and LN18 cell lines exhibit a slight but significant decrease of p62 levels, also called sequestosome 1 (SQSTM1), revealing an autophagy process (Fig 3D). Interestingly, bafilomycin A1 treatment was also able to slightly reverse the metformin effect on GB cell death, suggesting that autophagy processes could contribute to the metformin-induced cell death phenotype (S3A Fig). These observations indicate that metformin treatment in human glioma cells induces autophagy, which might also participate to anti-cancer effects of metformin.

### AMPK and Redd1/DDIT4 only partially contributes to metformin anti-proliferative effects

**Fig 4:**
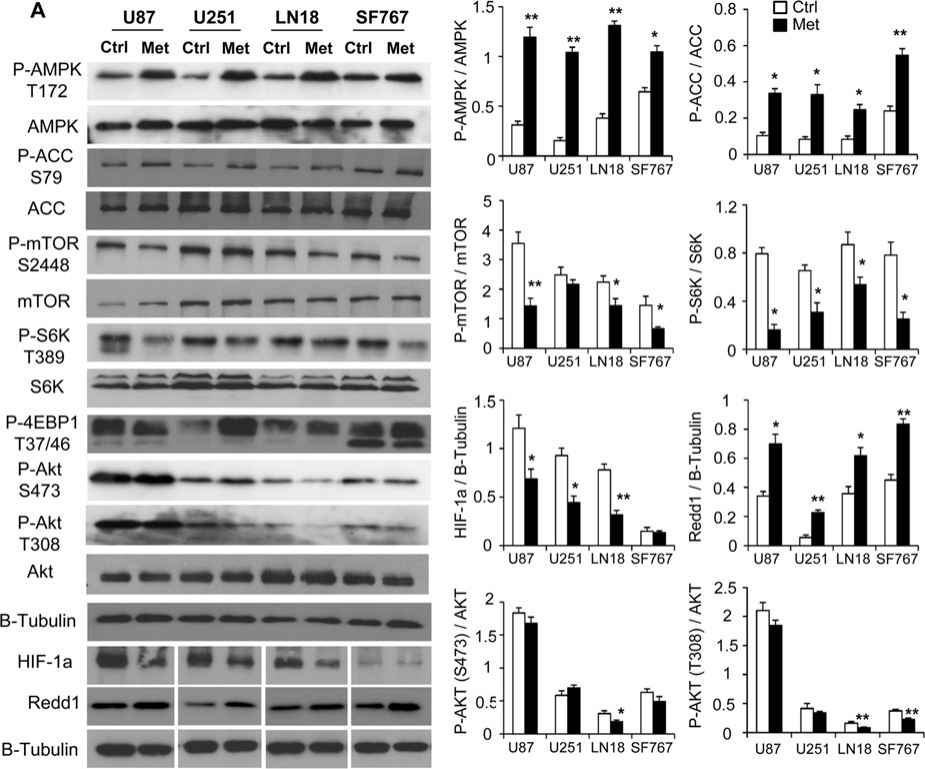
AMPK and mTOR pathways are modulated in response to metformin in GB cells. (**A**) Western Blot analyses of AMPK and mTOR pathways in U87, U251, LN18 and SF767 cells 48hrs after metformin treatment. Metformin increases AMPK activation leading to increased Acetyl-CoA Carboxylase (ACC) phosphorylation and decreases mTOR/AKT signaling leading to decreased S6K phosphorylation and 4EBP1 hypophosphorylation. Metformin also increases Redd1 expression and decreases HIF-1α expression (*p<0.05, **p < 0.01 compared to control, n = 3).

We then aimed to investigate the molecular mechanisms involved in the response to metformin in human GB cells. Numerous studies have previously demonstrated that metformin’s inhibition of mitochondrial ETCI and subsequent increase of the AMP/ATP ratio could activate AMPK leading to numerous downstream effects such as inhibition of mTOR [19, 31]. After 48hrs of metformin treatment, all glioma cells exhibit an activation of AMPK characterized by an increased phosphorylation at the Thr-172 site (Fig 4A). AMPK activation also correlates with the increased phosphorylation at Ser-79 of AMPK target, acetyl-CoA carboxylase (ACC), in the four GB cells. Next, we assessed the downstream effector of AMPK, mTOR and its pathway. We observed that metformin induces a decrease in Ser-2248 phosphorylation of mTOR (Fig 4A). This decreased activation state of mTOR was accompanied by decreased phosophorylation of its downstream targets, S6K and 4EBP1, at Thr-389 and Thr-37/46 (Fig 4A), respectively, suggesting a diminished activation of the mTOR pathway in GB cells following metformin treatment. Metformin has been shown to exert antiproliferative activity on Glioma Stem Cells (GSC), through inhibition of the AKT pathway, which is suggested to be an intracellular target of this drug [32]. Interestingly, in GB cell lines with WT PTEN (LN18 and SF767), where AKT can be regulated, we show that AKT phosphorylations (S473 and T308) were significantly inhibited in response to metformin treatment. In mutated PTEN GB cells (U87 and U251), AKT phosphorylations remained high and unaffected by metformin (Fig 4A). We also noted decreased HIF-1α levels and increased expression of Redd1/DDIT4 (Fig 4A), a stress-activated protein, which is down regulated in a subset of human cancers and also controls mTOR complex activity. Indeed, Redd1 can inhibit mTOR activity through activation of TSC2 [33], suggesting a possible role for Redd1 in metformin anti-cancer effects in human GB cells.

**Fig 5:**
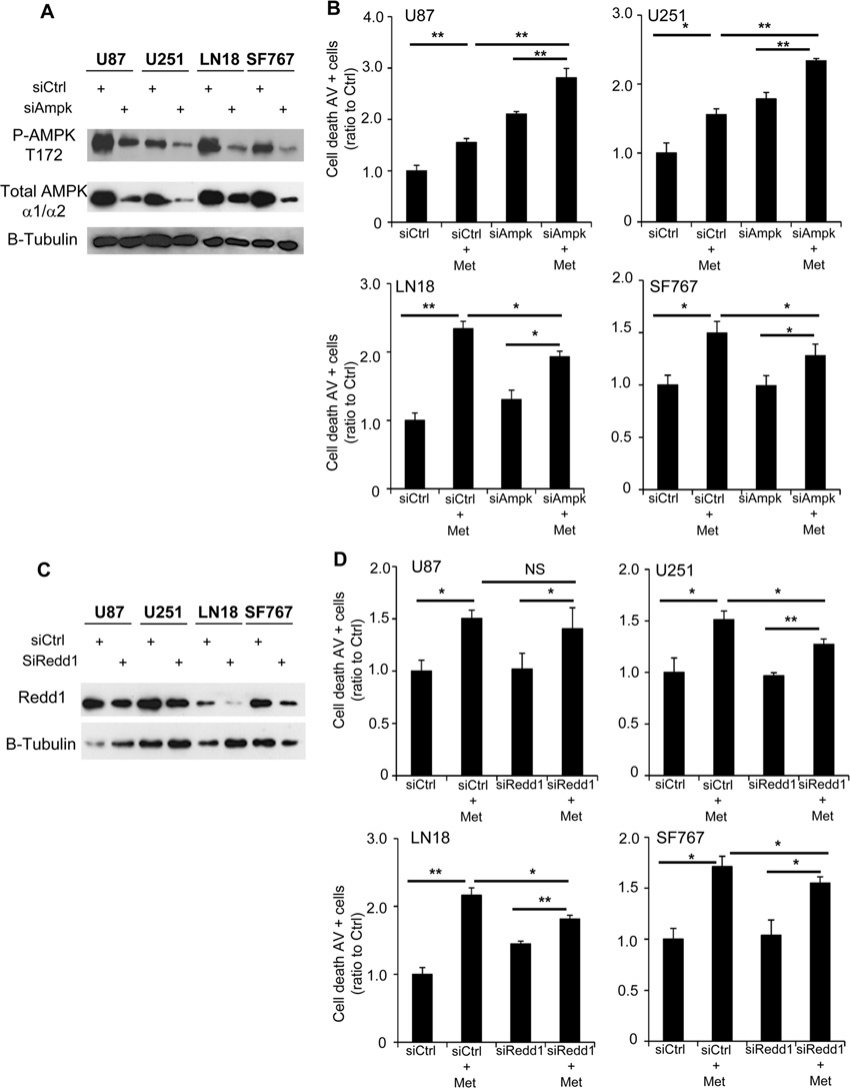
AMPK and Redd1 partially contribute to metformin-induced GB cell death. (**A**) Western Blot analyses showing AMPK inhibition after specific siRNA transfection. (**B**) Quantification of apoptotic and necrotic cell death, using flow cytometry and Annexin-V/PI staining, 48hrs after metformin treatment and with or without AMPK inhibition. AMPK inhibition increases cell death for U87 and U251 cells and slightly inhibits metformin effect on GB cell death. (*p<0.05, **p<0.01, n = 3). (**C**) Western Blot analyses showing Redd1 inhibition after specific siRNA transfection. (**D**) Quantification of apoptotic and necrotic cell death, using flow cytometry and Annexin-V/PI staining, 48hrs after metformin treatment and with or without Redd1 inhibition. Redd1 inhibition slightly inhibits metformin effect on GB cell death (*p<0.05, **p<0.01, n = 3).

As metformin has also been reported to exert anti-glioma effects independently of AMPK, including a recent study demonstrating that metformin enhances binding of PRAS40 with RAPTOR protein resulting in mTOR inhibition and suppression of glioma cell proliferation, independently of AMPK [19], we wanted to determine the contribution of AMPK in our models using RNA interference strategy and previously validated siRNAs (Fig 5A) [22]. In a similar way, we also tested the contribution of Redd1 in our GB cell lines (Fig 5C). As shown above, 48hrs of metformin treatment significantly increases cell death in all GB cells (siCtrl+Met) compared to the vehicle-treated control cells (siCtrl). Interestingly, AMPK inhibition (siAmpk) alone also increases cell death for U87 and U251 cells but not LN18 and SF767 cells, suggesting AMPK expression is important for cell survival in the basal state for some glioma cells (Fig 5B and S4 Fig). Following pretreatment with siAMPK, metformin still induced a significant level of apoptosis in all 4 GB cell lines (siAMPK+Met *versus* siAMPK alone). However, when comparing the effect of metformin in siAMPK-pretreated cell lines *versus* siCtrl-pretreated cell lines, knockdown of AMPK partially, but incompletely, abrogates the induction of apoptosis by metformin in all cell lines (Fig 5B). This suggests that metformin has both AMPK-dependent (LN18 and SF767) and AMPK-independent (U87 and U251) effects. Unlike inhibition of AMPK, Redd1 inhibition with siRedd1 did not induce increased GB cell death. Yet, similarly to siAMPK, metformin, following pretreatment with siRedd1, still induced a significant level of apoptosis in our GB cells (Fig 5D). Again, when comparing the effect of metformin in siRedd1-pretreated cell lines *versus* siCtrl-pretreated cell lines, knockdown of Redd1, as AMPK knockdown, partially, but incompletely, abrogates apoptosis induction at least in U251, LN18 and SF767 cells (Fig 5D). These results suggest that metformin effects are partially mediated by Redd1 in at least U251, LN18 and SF767 as well as AMPK in LN18 and SF767 cells but not in U87 and U251 cells.

### Metformin inhibits tumor growth *in vivo*

**Fig 6:**
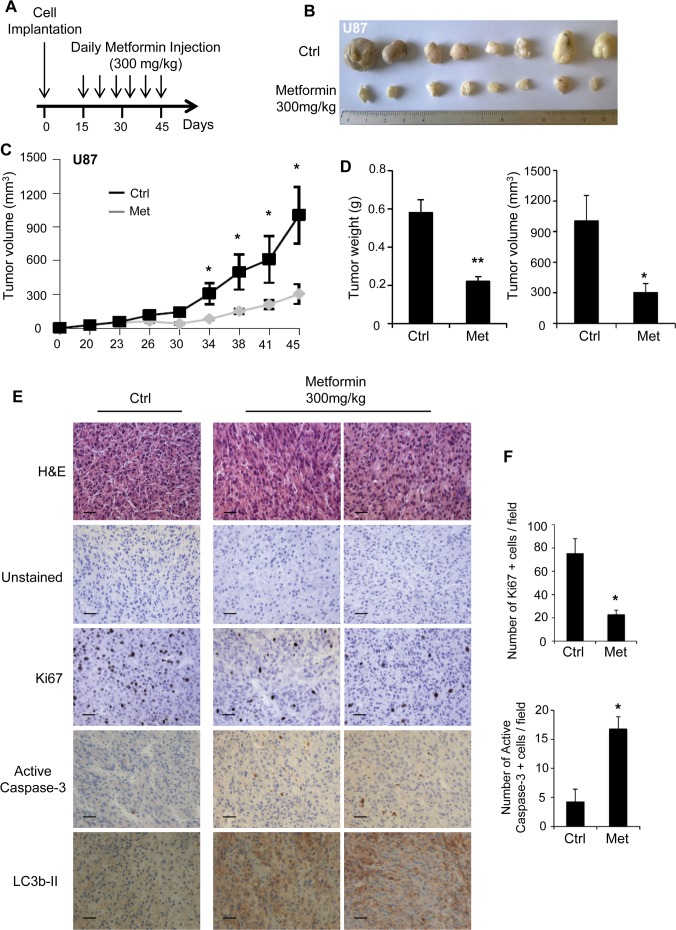
Metformin delays tumor growth *in vivo*. (**A**) After tumor formation, mice were treated daily with IP injection of metformin (300mg/kg). (**B**) Representative picture of tumors dissected from vehicle-treated control mice and metformin-treated mice 45 days post-GB cell implantation. (**C**) Graph representing U87 tumor growth in vehicle-treated control mice and metformin-treated mice. (**D**) Quantification of tumor weight and tumor volume 45 days post-GB cell implantation. Metformin significantly affects U87 tumor growth. (**E**) Representative unstained and H&E, Ki67, active caspase-3 and LC3b-II stained sections of U87 tumors grown in vehicle-treated mice and metformin-treated mice. Scale bars: 40μm. (**F**) Quantification of the number of Ki67 and active caspase-3 positive cells per field. Metformin treatment significantly decreases the number of proliferative cells and increases the number of apoptotic cells. (*p<0.05, **p<0.01 Met compared to Ctrl, n = 5 mice (10 tumors) per group).

Inhibition of tumor growth *in vivo* by metformin has been demonstrated in several cancer types [22, 34, 35]. However, few studies have highlighted the anti-neoplastic effect of metformin in glioma *in vivo*. To investigate this aspect, we injected immuno-deficient mice subcutaneously with U87 and LN18 cells. Following tumor formation, about 15 days after cell injection, we started to treat the mice daily with either vehicle or metformin (300mg/kg) [22] for 30 days and assessed tumor growth (Fig 6A). As shown in Fig 6 and S5 Fig, mice treated with metformin exhibit smaller tumors, in both U87 and LN18 xenografts, compared to vehicle-treated mice (Fig 6B and S5B Fig). Additionally, we measured tumor volume throughout the course of the study and we demonstrate that U87 and LN18 tumor growths were markedly reduced in metformin-treated mice (Fig 6C and S5A Fig). In line with these results, tumor weight and volume were significantly diminished by metformin treatment at the end of the study (Fig 6D and S5C Fig). Immunohistochemistry experiments performed on sections of U87 or LN18 tumors from mice treated with metformin show a decrease in Ki67 staining and an increase in active caspase-3 and LC3b-II staining compared to tumors from vehicle-treated mice (Fig 6E and S5D Fig). Specifically, quantification of Ki67 and active caspase-3 positive cells reveals that metformin treatment significantly reduces tumor growth and cell proliferation (Ki67) by 3.5-fold and 2-fold within U87 and LN18 tumors, and increases tumor cell death (active caspase-3) by 4-fold and 6-fold within U87 and LN18 tumors, respectively (Fig 6F and S5E Fig). These results confirm that metformin significantly reduces tumor growth *in vivo*.

### Metformin enhances the effect of temozolomide and irradiation

**Fig 7:**
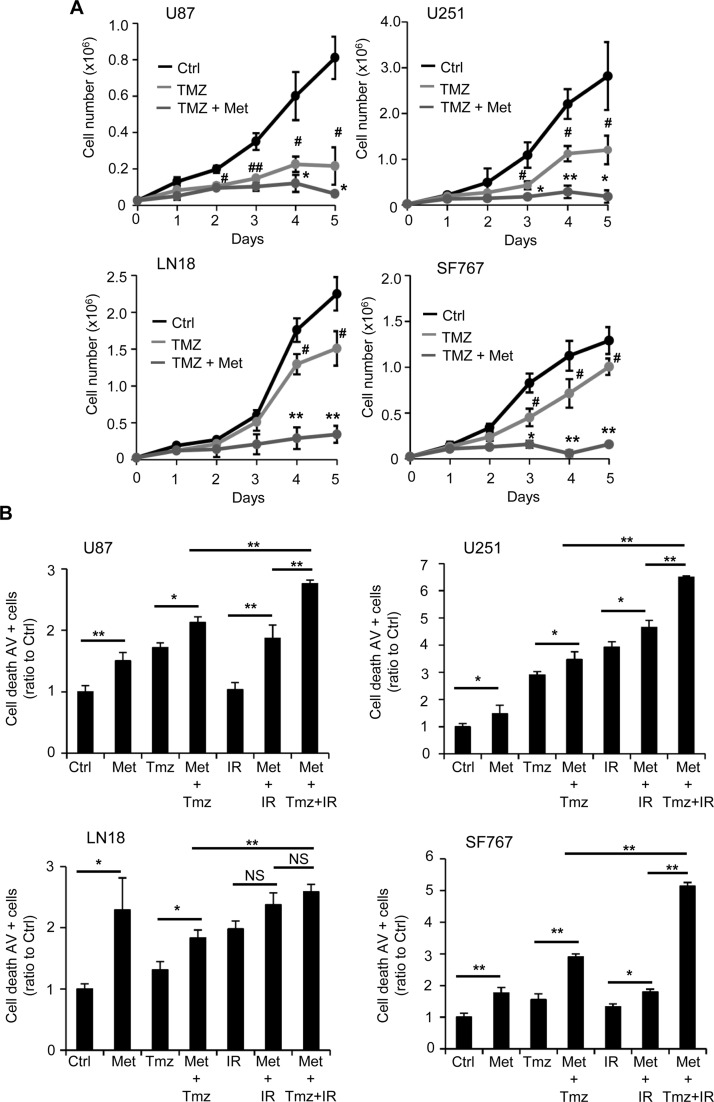
Metformin enhances the effect of temozolomide and irradiation on GB cells. (**A**) Proliferation assays performed with U87, U251, LN18 and SF767 cells show that metformin enhances the anti-proliferative effect of temozolomide (TMZ) (black curve, Ctrl: PBS vehicle control; light grey curve, TMZ: temozolomide 10μM (U87), 5μM (U251), 50μM (LN18), and 30μM (SF767); dark grey curve, TMZ+Met: temozolomide 10μM (U87), 5μM (U251), 50μM (LN18), and 30μM (SF767) and metformin 10mM) (#p<0.05, ##p<0.01, TMZ vs. Ctrl, *p<0.05, **p<0.01, TMZ+Met vs. TMZ, n = 4). (**B**) Quantification of cell death, using flow cytometry and AV/PI staining, 48hrs after metformin treatment and/or TMZ and/or irradiation (IR, 5Gy). TMZ and/or IR combined with metformin present a stronger effect on cell death than TMZ or IR alone, particularly in U87, U251 and SF767 GBM cells (*p<0.05, **p<0.01, n = 4).

Finally, an expanding body of evidence indicates that combining drugs targeting cell metabolism, with chemotherapeutic agents or radiotherapy is becoming an attractive therapeutic option in cancer [8, 9, 36, 37]. In these perspectives, we wondered whether or not metformin could represent a potential enhancer of the cytotoxic effects of temozolomide (TMZ) and/or radiotherapy. Accordingly, we performed a proliferation assay in which U87, U251, LN18 and SF767 GB cells were treated or not with 10mM metformin and an appropriate dose of temozolomide, 10μM, 5μM, 50μM and 30μM, respectively (Fig 7A and S6 Fig). In this experiment, we used different concentrations of chemotherapeutic agent due to the resistance status and sensitivity to TMZ of each glioma cell line. TMZ damages can be repaired by MGMT, which induces treatment resistance and methylation of the MGMT promoter leads to increased sensitivity [6]. Among the cell lines we used, U87 and U251 exhibit MGMT methylation and higher sensitivity to TMZ compared to LN18 and SF767, where the MGMT promoter is not methylated (S1A Fig). As shown in Fig 7A and S6A Fig, the appropriate dose of TMZ induces a significant decrease in all GB cell proliferation, starting at 48-72hrs after treatment. As expected, U87 and U251 cells show an increased sensitivity to TMZ due to their MGMT status compared to LN18 and SF767 cells. Interestingly, when we combined temozolomide and metformin (TMZ+Met), we were able to achieve a stronger and significant anti-proliferative effect than with TMZ alone. Cell counts were particularly decreased in LN18 and SF767 cells treated with TMZ and metformin compared to TMZ conditions, suggesting a particular proliferative sensitization by metformin in normally TMZ-resistant cells (Fig 7A and S6A Fig). To further analyze the effects of TMZ or TMZ+Met treatments in our GB cells, we looked more specifically at the GB cell death mechanism in response to temozolomide (TMZ), metformin and temozolomide (Met+TMZ), irradiation (IR), irradiation and metformin (Met+IR) and temozolomide, irradiation and metformin (Met+TMZ+IR), using Annexin-V/PI staining and flow cytometry (Fig 7B and S7A Fig). U87, U251, LN18 and SF767 GB cells were treated with either TMZ (100μM), Met (10mM)+TMZ (100μM), IR (5Gy), Met (10mM)+IR (5Gy) or combination of Met (10mM)+TMZ (100μM)+IR (5Gy). These specific TMZ and irradiation doses were used in order to induce GB cell death. Forty eight hours following treatments, we consistently observed a significant increase in cell death with all treatments compared to control vehicle-treated/non-irradiated cells (Fig 7B and S7A Fig). Moreover, we observed that both combinations TMZ or IR with metformin induced significantly more cell death than the respective treatment alone (TMZ or IR) at least in U87 (Met+TMZ: 19.1% of Annexin-V+ cells vs TMZ: 12.1%; Met+IR: 25% vs IR: 10.8%), U251 (Met+TMZ: 29.4% vs TMZ: 19.6%; Met+IR: 39.4% vs IR: 33.2%) and SF767 (Met+TMZ: 14.60% vs TMZ: 7.81%; Met+IR: 11.2% vs IR: 7.3%) cell lines (Fig 7B and S7A Fig). Additionally, full combination of metformin with TMZ and IR (Met+TMZ+IR) clearly induces more cell death than the respective single or dual tested combination (Fig 7B and S7A Fig). Altogether, these results suggest that combining metformin with TMZ or radiotherapy could potentially enhance the efficacy of these therapies against human glioma cells.

## Discussion

Developing new therapeutic strategies against GB is critical for these aggressive brain tumors, and combining drugs targeting cell metabolism with chemotherapeutic agents or radiotherapy is an attractive approach [2, 38]. In the present study, we were particularly interested in a biguanide molecule used for type 2 diabetes treatment, metformin. Metformin is a hydrophobic drug, which requires organic cation transporters, is known to easily cross the blood-brain barrier [39] and has already been shown to inhibit the growth of leukemic, pancreatic, colon, prostate, ovarian and breast cancer cells leading to numerous clinical trials [14, 40]. Studies have already shown that patients with breast cancer treated with a combination of chemotherapy and metformin have higher pathological response rates compared to those treated with chemotherapy alone. However, few studies highlighted the efficacy of metformin and its use in combination with radio/chemotherapy as an anti-glioma agent. Interestingly, most recent studies have shown that metformin affects GSC proliferation, self-renewal capacities and survival with a higher efficiency compared to differentiated glioma cells. The pathways involved in GSC are still unclear but it is suggested that AKT and mTOR could play a role in the observed phenotypes [19, 32]. It has also been demonstrated that metformin induces GSC differentiation through a FOXO3-AMPK dependent mechanism [18]. In differentiated GB cells, it has been previously demonstrated that metformin suppresses U251 cell adhesion and invasion through the inhibition of fibulin-3 expression [41].

Our present work and other recent studies report that metformin markedly reduces human glioma cell proliferation. Although metformin impacts cell proliferation, we observed that replacing cell media daily with fresh media partially abrogated the effect of metformin on cell proliferation. These results suggest that metformin-induced toxicity also results from off-target effects, which could be due to increased glycolytic pathway activity in cells treated with metformin. This metabolic shift favors lactate production leading to media acidification and toxicity, and/or higher glucose consumption leading to glucose depletion and cell starvation over the experimental time course, as shown in previous reports [22, 42]. Of note, LN18 and SF767 cell proliferation was more affected than U87 and U251 cells in both culture conditions. Both LN18 and SF767 cell lines are wild-type (WT) for PTEN and present functional PTEN expression that can oppose PI3K signaling leading to inactivation of the AKT survival pathway, or at least some modulation in AKT activation and decrease of glucose consumption and Warburg effect (S1 Fig). Metformin has been shown to decrease AKT activity in breast cancer cells and in glioma stem cells through insulin-receptor signaling, which is another potential mechanism for metformin’s anti-neoplastic activity [32, 43, 44]. Thus, a possible explanation for the higher sensitivity to metformin for LN18 and SF767 cells could be that metformin down-regulates AKT in a more effective way in these PTEN WT cells compared to PTEN mutated cells (U87, U251) where the PI3K/AKT pathway is constitutively active. Indeed, initial evidence reported in our study show that AKT phosphorylations at sites S473 and T308 were significantly inhibited especially in PTEN WT LN18 and SF767 cells as compared to PTEN mutated U87 and U251 cells. These data lead us to hypothesize that PTEN status could represent a good criterion to determine GB cell sensitivity to metformin treatment as metformin could only inhibits AKT phosphorylations when AKT activation is not constitutive due to PTEN mutation.

Metformin treatment reduces the number of GB cells undergoing cell division and increases cell cycle arrest leading to decreased cell proliferation. We further demonstrated that cell death and apoptotic processes occurred in our GB cells following metformin treatment confirming previous studies in B16 melanoma and Acute Myeloid Leukemia (AML) cells [22, 28]. Time course experiments allowed us to clarify the timeline of the different processes induced in response to metformin treatment in our GB cells and suggest that metformin would first induce cell cycle arrest followed by autophagic and cell death processes. Autophagy is considered as a survival mechanism induced in adverse conditions to maintain cell integrity, but paradoxically, it is also involved in cell death if the adverse conditions are persistent [45]. Indeed, recent works demonstrated that autophagy was primarily responsible for the anti-proliferative effects of metformin in melanoma and lymphoma cells through mTOR pathway inhibition [28, 31]. Additionally, they demonstrated that AMPK siRNA partially prevented cell death induced by metformin suggesting that metformin induces autophagy and anti-cancer effects in melanoma cells with both AMPK-dependent and AMPK-independent pathways [28, 34]. Interestingly, treatment with bafilomycin A1, an autophagy inhibitor, was able to partially rescue the cell death phenotype observed in response to metformin treatment suggesting that metformin-induced persistent autophagy could lead to cell death in our GB cell lines.

The molecular mechanisms involved in the anti-cancer activity of metformin are not yet clearly understood, particularly in glioma. Some studies have reported that metformin exhibits its activity through a block in G_0_/G_1_ cell cycle progression, induction of cell death associated with JNK activation, mitochondrial membrane depolarization and oxidative stress [16]. These mechanisms have been shown to be AMPK dependent and/or independent. A recent work demonstrates that metformin inhibits mitochondrial respiration, without affecting ATP levels in GSCs, and mTOR pathway through an AMPK-independent enhancement of PRAS40-RAPTOR association to suppress GB cell growth [19]. In our four different glioma cell lines, we demonstrate, using Seahorse technology, that metformin efficiently inhibits mitochondrial respiration. These data are consistent with previous report showing that metformin inhibits mitochondrial complex I leading to a significantly decreased respiration in breast and colon cancer cells and diminished tumorigenesis [43, 46]. Furthermore, we show that metformin significantly inhibits mitochondrial ETCI in our GB cells. However, this inhibition is surprisingly not as complete as we expected. This suggests that ETCI inhibition may not need to be complete to induce downstream effects or it is possible that metformin’s mechanism of action in GB cells involves additional processes other than ETCI inhibition. Interestingly, Madiraju et al. recently suggested that we may need to look beyond complex I at other mitochondrial enzymes. In fact, this group determined that metformin non-competitively inhibits the redox shuttle enzyme mitochondrial glycerophosphate dehydrogenase in both rat and human mitochondrial lysates [47]. Altogether, our data suggests that mitochondria are the primary target of metformin but the exact mitochondrial target remains in a controversial standing. Although some works reported that metformin does not always affect cellular ATP to induce an energetic stress [19], it is established that decreased mitochondrial respiration can lead to a reduction in ATP production. Our data indicate a significant decrease in global ATP in our differentiated GB cells treated with metformin. Despite compensatory mechanisms, such as increased glycolytic ATP production, this could lead to an energetic stress, which combined with additional effects of metformin, could result in glioma cell growth inhibition. GSCs and more differentiated cells are likely to have a wide range of different metabolic properties as well as expression of uptake and extrusion proteins used in metformin accumulation, which could explain the different sensitivity to the drug [48].

While it is well established that metformin can activate AMPK, emerging evidence shows that metformin may modulate cancer activities, and particularly the mTOR pathway, through AMPK-independent mechanisms [11, 19, 49]. In our models, AMPK silencing partially, but incompletely, abrogates the cell death induced by metformin in GB cell lines. This suggests that the effects of metformin could be both AMPK-dependent, especially in LN18 and SF767 cells and AMPK-independent in U87 and U251 cells. This siAMPK data, however, is difficult to interpret due to the facts that siAMPK alone induces significant cell death in U87 and U251 cells. Additionally, western blot analysis of the four GB cell lines following siAMPK pretreatment shows a significant, but incomplete, decrease of AMPK expression. Thus, it is certainly possible that the low residual AMPK expression is sufficient to mediate the metformin effect on cell death and therefore we would not expect to see a complete abrogation of the effect of metformin. In addition to AMPK modulation, we also found that metformin treatment in human glioma cell induces a significant increase in Redd1/DDIT4 expression, as previously reported for prostate cancer cells [50]. Studies have reported that Redd1 may be activated by energy and environmental stress, such as ATP depletion [51], starvation [52] and high cell density [53]. Interestingly, in neuronal PC12 cells, Redd1 expression was toxic as it increased sensitivity to ischemic injury and oxidative stress [54]. It is also known that Redd1 overexpression is enough to potently inhibit mTOR activity, while loss of Redd1 reduces the ability of hypoxia to inhibit mTOR, and cells with nonfunctional Redd1 are defective in downregulation of S6K, S6 and 4E-BP1 phosphorylation following energy depletion [51]. Interestingly, Redd1 inhibition was able to partially rescue the metformin-induced cell death phenotype in our treated GB cells suggesting that the modulation of Redd1 expression could potentially be another explanation to metformin anti-cancer effects in human GB cells. Additionally, and similar to siAMPK data, western blot analysis following siRedd1 treatment shows a significant, but incomplete, decrease of total Redd1 expression, which could be insufficient to counteract metformin effect on cell death. Although further investigations are required, this mechanism could represent another way for metformin to modulate the mTOR pathway in glioma cells. Additionally, the decreased HIF-1α expression accompanying the increased Redd1 expression can be correlated to the fact that like other proteins, such as p53 [55], it has been shown that increased Redd1 level leads to a decrease in HIF-1α expression [56]. Redd1 appears to decrease HIF-1α by translocating to mitochondria and directly decreasing mitochondrial ROS leading to destabilization of HIF-1α [56]. The decrease of HIF-1α expression in GBM cells is also another potential mechanism to explain diminished proliferation as it is now clearly established in glioma that HIF-1α inhibition induces a decreased proliferation and survival.

Our *in vitro* data led us to assess the efficiency of metformin *in vivo* in a mouse model of GB. We demonstrate that daily metformin treatment (300mg/kg) reduces both U87 and LN18 tumor growth (Fig 6 and S5 Fig). Of note, we were not able to grow U251 and SF767 cells in our immunodeficient mouse model. The concentration of metformin we administered intraperitoneally to the mice did not induce any toxicity and is within the range of concentrations (50–400 mg/kg) traditionally used for *in vivo* experiments with metformin. Although the dose is higher than what is typically given to diabetic patients (500–2500 mg metformin per day) [57], it is important to recognize that the metformin dose given to diabetic patients is not the maximum possible dosage, but the minimum dosage that provides adequate glycemic control. Additionally, diabetes treatment with metformin requires long-term therapy while treatment of glioma patients with metformin would be used for short time periods. We think that metformin concentration we used could be clinically achieved in patients and with limited side effects. We do, however, recognize that this concentration could only be clinically achieved in patients without liver failure to prevent complications. Similarly to the block in G_0_/G_1_ phase and the increased GB cell death that we observed in our cell lines, we found that metformin treatment significantly decreases Ki67-positive cells and increases active caspase-3-positive cells in tumors from treated animals. These results indicate that metformin induces GB cell cycle arrest and cell death *in vivo* and confirms the previously performed GSC orthotopic xenograft experiments showing that metformin clearly suppresses tumor-initiating potential of these GSCs [18, 19].

Finally, we aimed to determine the effect of metformin in combination with the standard treatment for GB, temozolomide (TMZ) and radiotherapy (IR). Using metformin as an adjunct to cancer chemotherapy is becoming a more and more attractive strategy [26, 44]. However few studies reported the effect of the combination of metformin and TMZ or radiations in cancer cells [58–60], and only one previous study reported that metformin combined with sorafenib, a RAS/RAF/MAPK pathway inhibitor, and TMZ induces a significant increase in toxicity on TMZ-resistant GSCs [61]. This data suggest that in GSCs, metformin could act as a pro-oxidant factor when combined with other drugs such as sorafenib or TMZ. An important determinant of TMZ failure is the presence of the MGMT enzyme, which removes the alkyl groups formed by TMZ. MGMT expression is associated with a limited benefit from TMZ and methylation of its promoter was linked to improved outcomes and is currently a promising molecular prognostic marker in the glioma field. Our study used human GB cells known for their methylation status of MGMT, and we show that combining metformin with TMZ and/or radiotherapy could potentially enhance the efficacy of these therapies even in resistant unmethylated MGMT cells such as SF767. Consistent with what is reported in the literature, metformin could act as a pro-oxidant factor and enhance the production of ROS increasing the oxidative stress induced by TMZ or radiotherapy treatment [7, 37, 61].

In summary, our data indicate that metformin reduces GB cell tumorigenesis. This metabolic agent induces GB cell cycle arrest, autophagic and apoptotic processes *in vitro* and *in vivo* potentially due to AMPK and Redd1 activation, mitochondrial energetic and mTOR pathway inhibition. We also demonstrate that metformin could act as a GB cell sensitizer/enhancer for TMZ treatment and/or radiotherapy. Introducing this promising new form of treatment may lead to an exciting and effective adjuvant therapy, which is directly needed for the treatment of glioblastoma.

## Supporting Information

S1 Fig Metformin inhibits GB cell proliferation. (**A**) Table showing the mutational status of the different glioblastoma cell lines. U87 and U251 cells are mutated for PTEN, U251 and LN18 cells are mutated for p53 and U87 and U251 cells present methylation of the MGMT promoter. (**B**) Representative photographs of U87, U251, LN18 and SF767 GB cells treated or not with metformin (10mM). Photographs were taken at day 5 of the proliferation assay (scale bars: 60 μm). (**C**) Proliferation assays performed with U87, U251, LN18 and SF767 glioma cells showing a decreased cell number in presence of metformin. In this experiment, cell media was replaced daily with fresh media containing or not metformin. (black curve, Ctrl: PBS vehicle control; grey curve, Met: metformin 10mM) (*p<0.05, **p<0.01, ***p<0.001 Met vs. Ctrl, n = 3).
(PDF)

S2 Fig Metformin affects different cell processes. (**A**) Representative plots of Ki67/PI stained U87, U251, LN18 and SF767 cells treated or not with metformin (10mM) for 48hrs. (**B**) Representative plots of Annexin-V/PI stained U87, U251, LN18 and SF767 cells treated or not with metformin (10mM) for 48hrs. (**C**) Representative plots of Acridine Orange stained U87, U251, LN18 and SF767 cells treated or not with metformin (10mM) for 48hrs.
(PDF)

S3 Fig Autophagy and metformin-induced GB cell death. (**A**) Quantification of apoptotic and necrotic cell death, using flow cytometry and Annexin-V/PI staining, 48hrs after metformin (10mM) treatment and with or without bafilomycin (10μM). Autophagy inhibition slightly reverses metformin effect on GB cell death. (*p<0.05, **p<0.01, n = 4).
(PDF)

S4 Fig AMPK inhibition and metformin in GB cells. (**A**) AMPK pathway slightly contributes to the metformin-induced GBM cell death. Representative plots of Annexin-V/PI stained U87, U251, LN18 and SF767 cells treated or not with metformin (10mM) for 48hrs and preliminarily transfected with a control (siCtrl, 500nM) or a specific AMPK (siAMPK, 500nM) siRNA.
(PDF)

S5 Fig Metformin delays tumor growth *in vivo*. (**A**) After tumor formation, mice were treated daily with IP injection of metformin (300mg/kg). Graph representing LN18 tumor growth in vehicle-treated control mice and metformin-treated mice. (**B**) Representative picture of tumors dissected from vehicle-treated control mice and metformin-treated mice 45 days post-GB cell implantation. (**C**) Quantification of tumor weight and tumor volume 45 days post-GB cell implantation. Metformin significantly affects LN18 tumor growth. (**D**) Representative unstained and H&E, Ki67, active caspase-3 and LC3b-II stained sections of LN18 tumors grown in vehicle-treated mice and metformin-treated mice. Scale bars: 40μm. (**E**) Quantification of the number of Ki67 and active caspase-3 positive cells per field. Metformin treatment significantly decreases the number of proliferative cells and increases the number of apoptotic cells (*p<0.05, **p<0.01 Met compared to Ctrl, n = 5 mice (10 tumors) per group).
(PDF)

S6 Fig Metformin enhances TMZ effect on GBM cells. (**A**) Representative photographs of U87, U251, LN18 and SF767 cells treated or not with temozolomide (respectively, 10μM, 5μm, 50μm and 30μm) and/or metformin (10mM). Photographs were taken 48hrs after treatment (scale bars: 40μm).
(PDF)

S7 Fig Metformin enhances TMZ and IR effects. (**A**) Representative plots of Annexin-V/PI stained U87, U251, LN18 and SF767 cells treated or not with temozolomide (TMZ, 100μm), and/or radiations (IR, 5Gy) and/or metformin (10mM) for 48hrs. TMZ and/or IR combined with metformin present a stronger effect on cell death than TMZ or IR alone, particularly in U87, U251 and SF767 cells.
(PDF)
