# Supplementary material for: Metformin Inhibits Growth of Human Glioblastoma Cells and Enhances Therapeutic Response
Source: PLoS One. 2015 Apr 13;10(4):e0123721. doi: 10.1371/journal.pone.0123721 (PMC4395104; doi:10.1371/journal.pone.0123721)

A

Cell line	PTEN status	p53 status	MGMT status
U87	Mutated	Wild-type	Methylated
U251	Mutated	Mutated	Methylated
LN18	Wild-type	Mutated	Non Methylated
SF767	Wild-type	Wild-type	Non Methylated

B

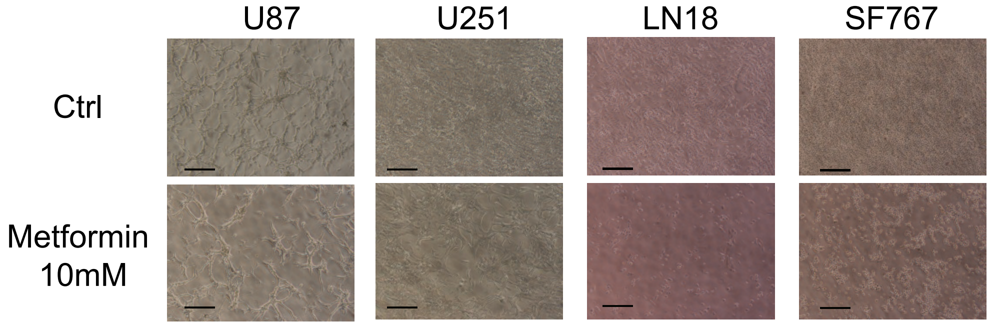

C

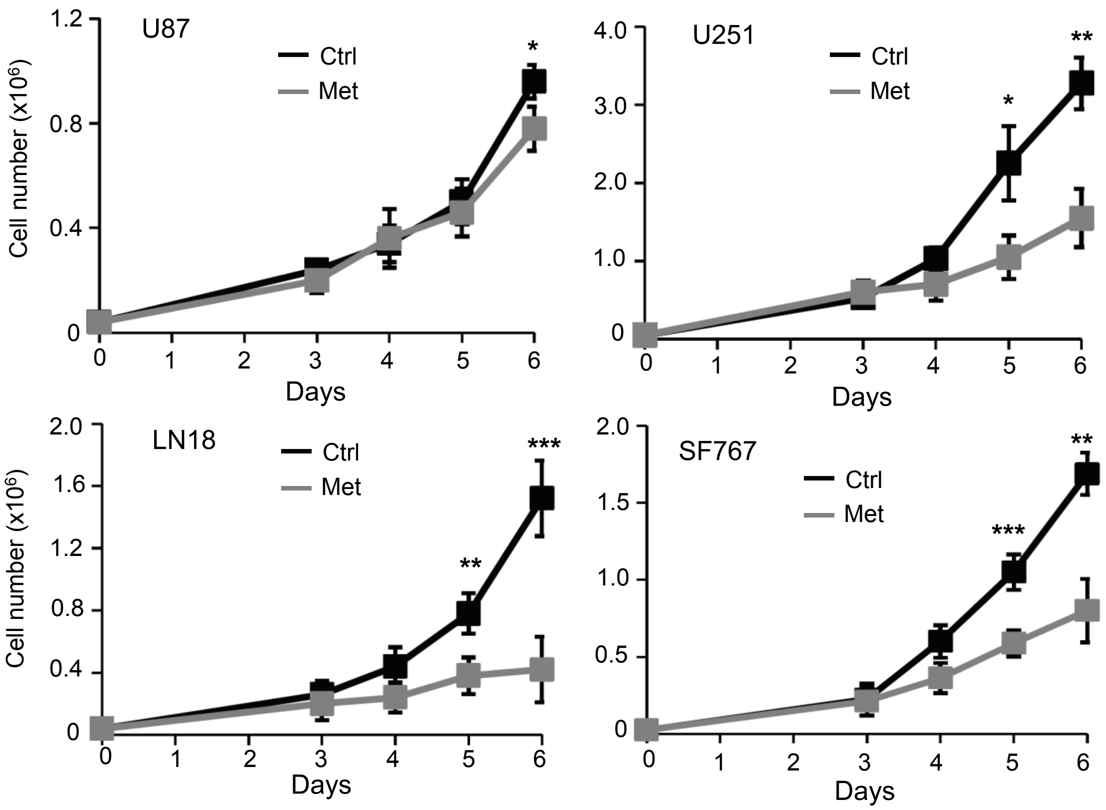

Supplement: S1 Fig — (A) Table showing the mutational status of the different glioblastoma cell lines. U87 and U251 cells are mutated for PTEN, U251 and LN18 cells are mutated for p53 and U87 and U251 cells present methylation of the MGMT promoter. (B) Representative photographs of U87, U251, LN18 and SF767 GB cells treated or not with metformin (10mM). Photographs were taken at day 5 of the proliferation assay (scale bars: 60 μm). (C) Proliferation assays performed with U87, U251, LN18 and SF767 glioma cells showing a decreased cell number in presence of metformin. In this experiment, cell media was replaced daily with fresh media containing or not metformin. (black curve, Ctrl: PBS vehicle control; grey curve, Met: metformin 10mM) (*p<0.05, **p<0.01, ***p<0.001 Met vs. Ctrl, n = 3). (PDF) [file pone.0123721.s001.pdf]
