# Supplementary figures and images for: Metformin Inhibits Growth of Human Glioblastoma Cells and Enhances Therapeutic Response
Source: PLoS One. 2015 Apr 13;10(4):e0123721. doi: 10.1371/journal.pone.0123721 (PMC4395104; doi:10.1371/journal.pone.0123721)

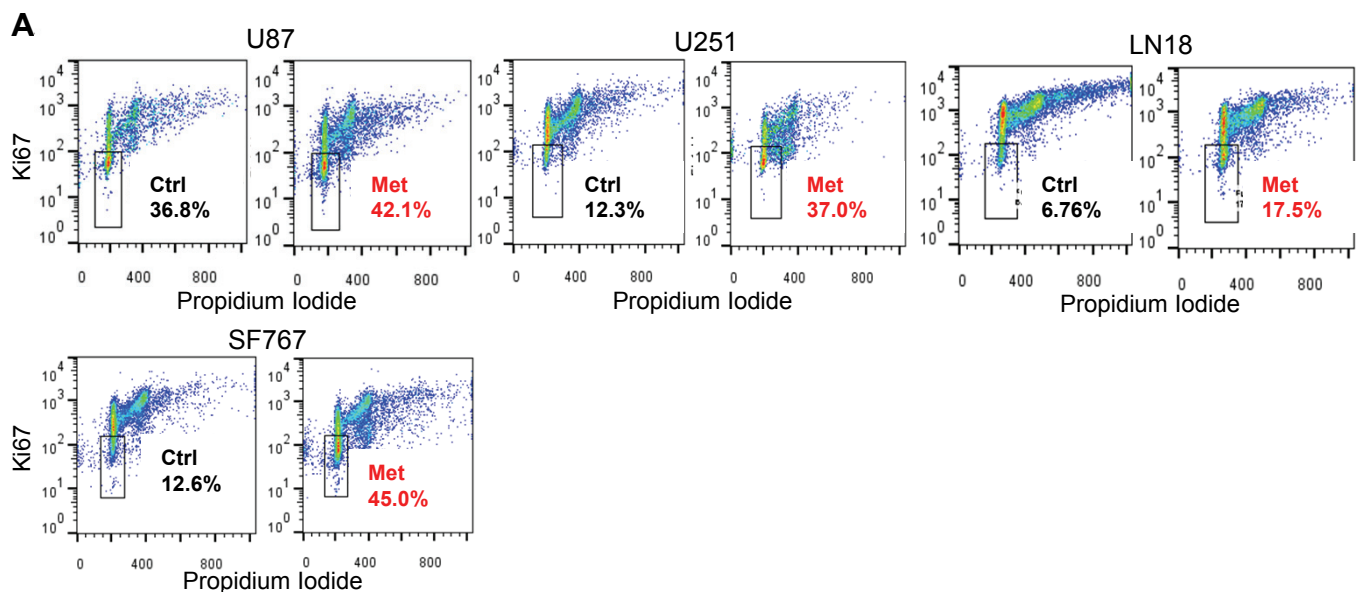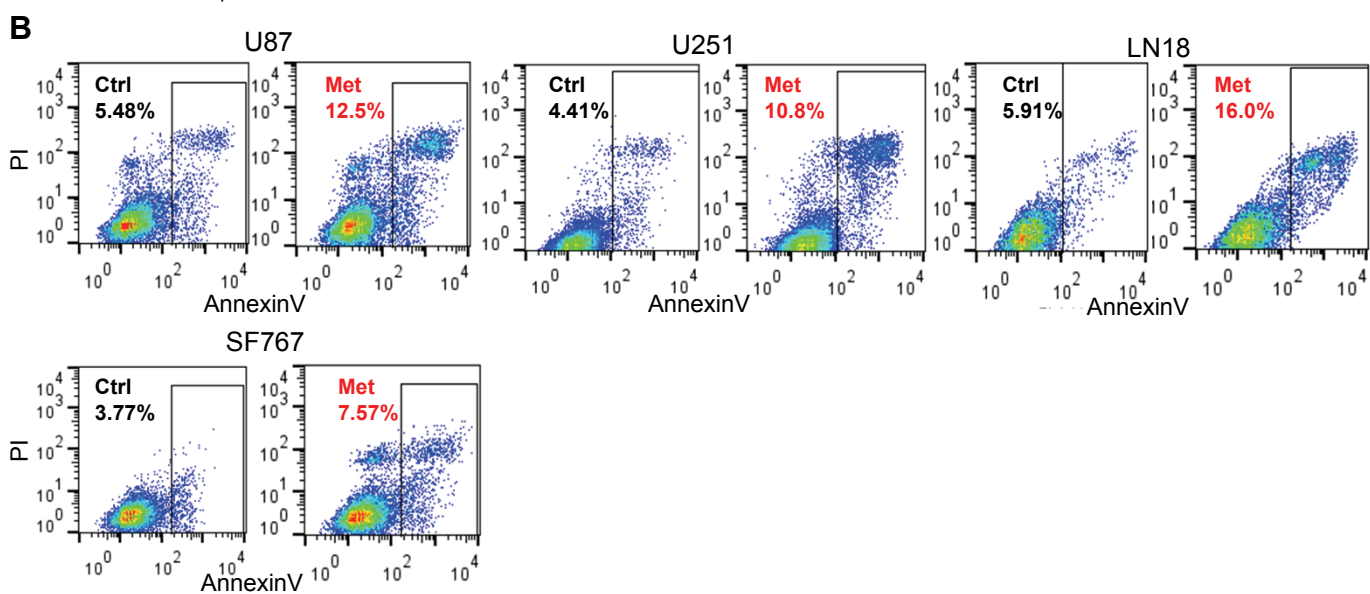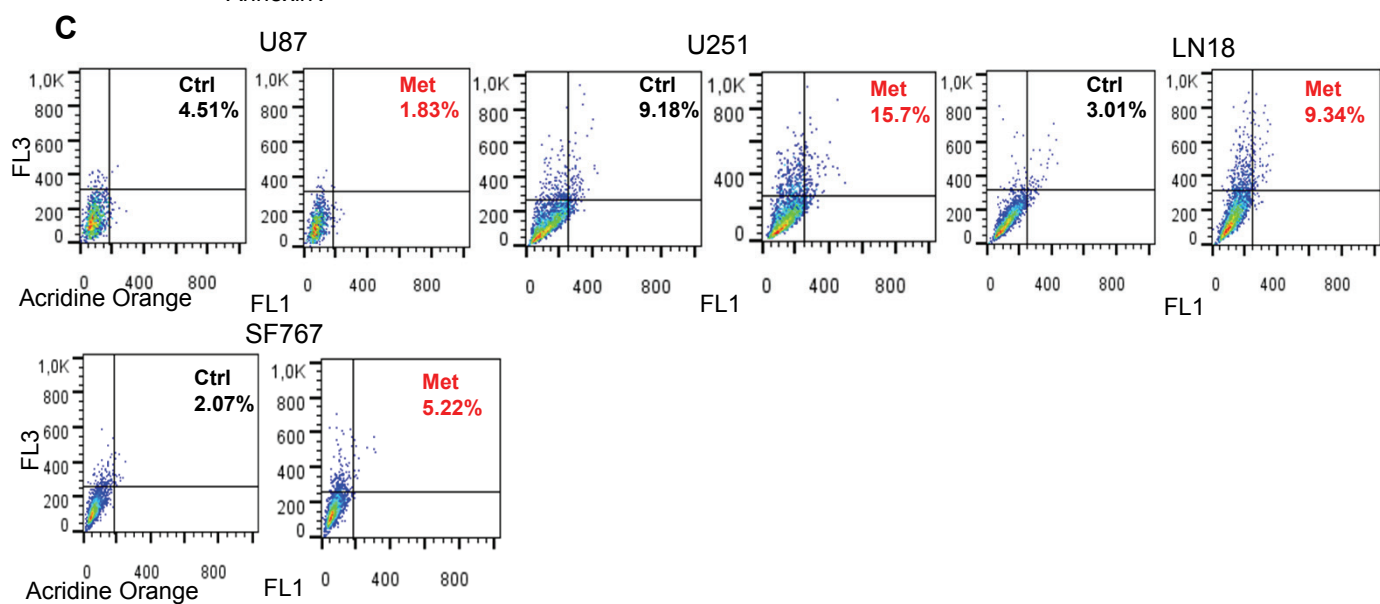

Supplement: S2 Fig — (A) Representative plots of Ki67/PI stained U87, U251, LN18 and SF767 cells treated or not with metformin (10mM) for 48hrs. (B) Representative plots of Annexin-V/PI stained U87, U251, LN18 and SF767 cells treated or not with metformin (10mM) for 48hrs. (C) Representative plots of Acridine Orange stained U87, U251, LN18 and SF767 cells treated or not with metformin (10mM) for 48hrs. (PDF) [file pone.0123721.s002.pdf]

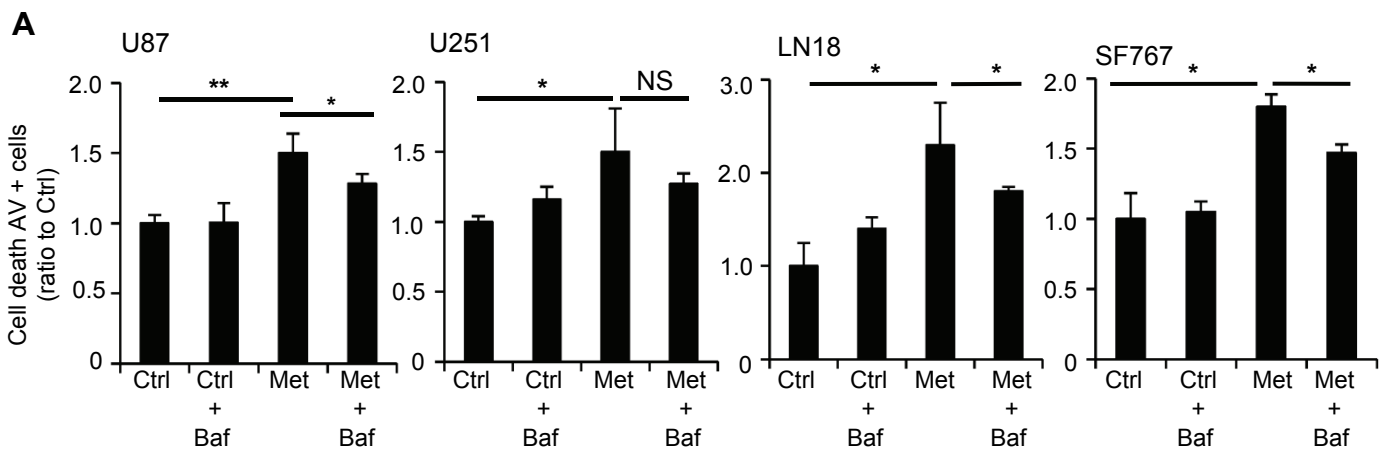

Supplement: S3 Fig — (A) Quantification of apoptotic and necrotic cell death, using flow cytometry and Annexin-V/PI staining, 48hrs after metformin (10mM) treatment and with or without bafilomycin (10μM). Autophagy inhibition slightly reverses metformin effect on GB cell death. (*p<0.05, **p<0.01, n = 4). (PDF) [file pone.0123721.s003.pdf]

**A**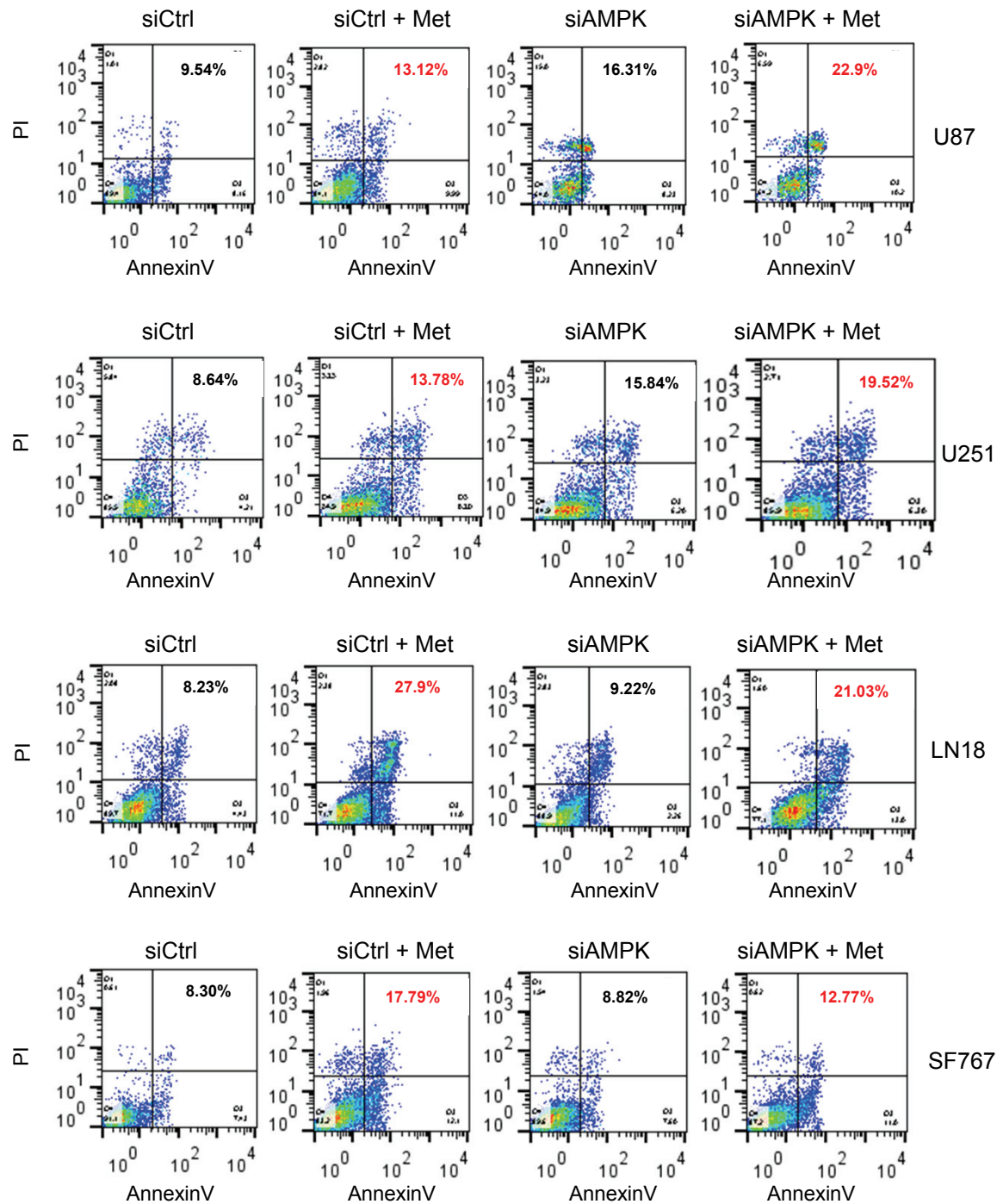

Supplement: S4 Fig — (A) AMPK pathway slightly contributes to the metformin-induced GBM cell death. Representative plots of Annexin-V/PI stained U87, U251, LN18 and SF767 cells treated or not with metformin (10mM) for 48hrs and preliminarily transfected with a control (siCtrl, 500nM) or a specific AMPK (siAMPK, 500nM) siRNA. (PDF) [file pone.0123721.s004.pdf]

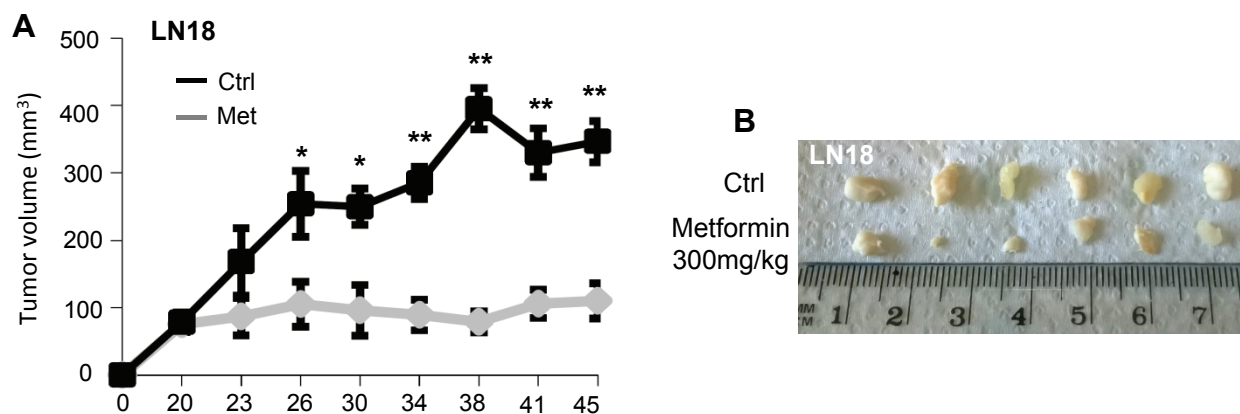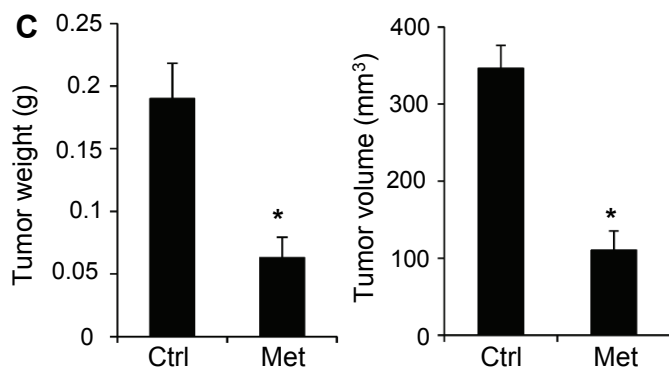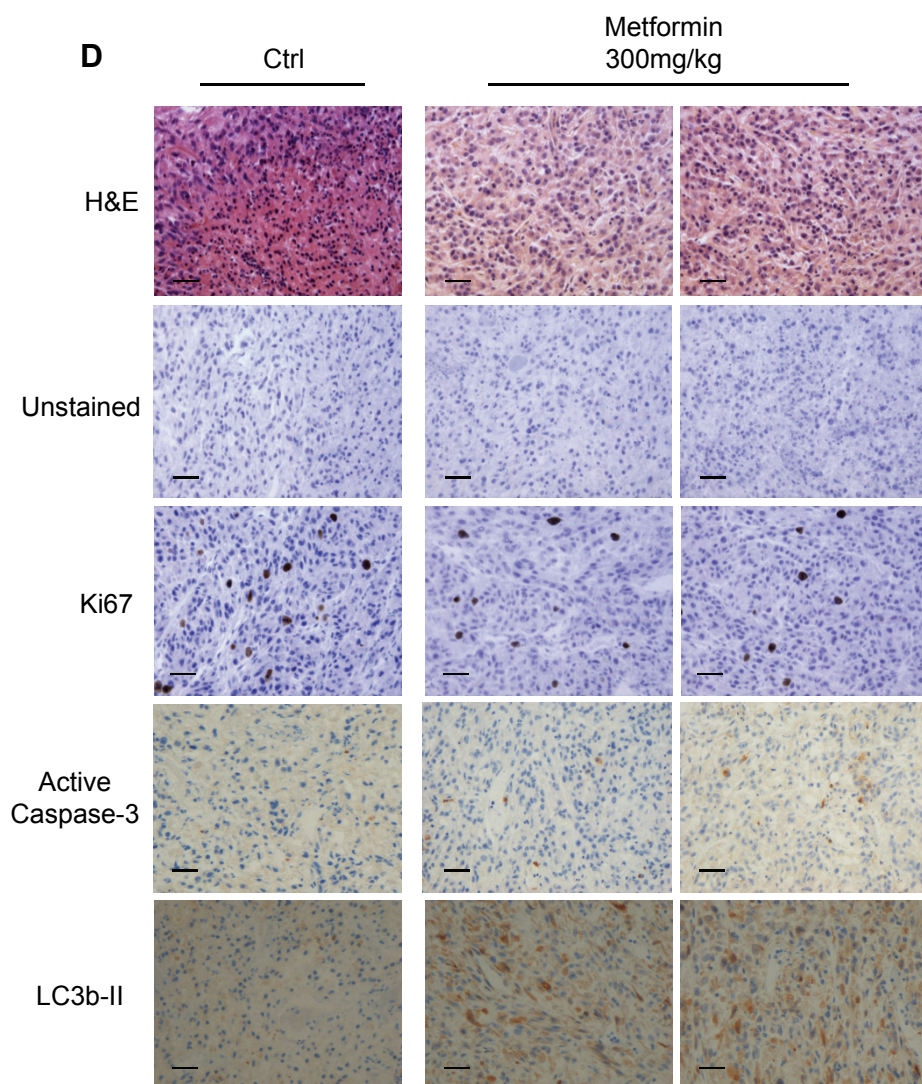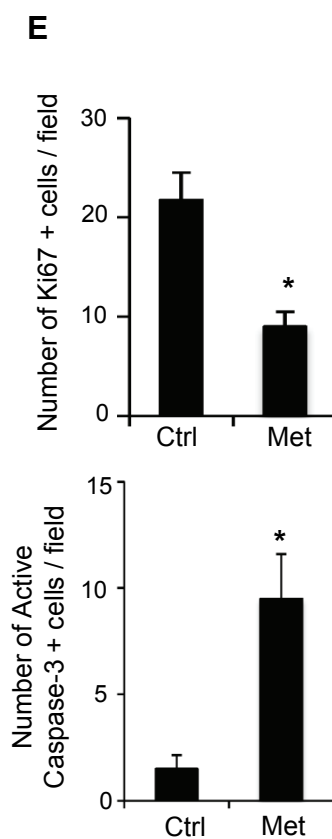

Supplement: S5 Fig — (A) After tumor formation, mice were treated daily with IP injection of metformin (300mg/kg). Graph representing LN18 tumor growth in vehicle-treated control mice and metformin-treated mice. (B) Representative picture of tumors dissected from vehicle-treated control mice and metformin-treated mice 45 days post-GB cell implantation. (C) Quantification of tumor weight and tumor volume 45 days post-GB cell implantation. Metformin significantly affects LN18 tumor growth. (D) Representative unstained and H&E, Ki67, active caspase-3 and LC3b-II stained sections of LN18 tumors grown in vehicle-treated mice and metformin-treated mice. Scale bars: 40μm. (E) Quantification of the number of Ki67 and active caspase-3 positive cells per field. Metformin treatment significantly decreases the number of proliferative cells and increases the number of apoptotic cells (*p<0.05, **p<0.01 Met compared to Ctrl, n = 5 mice (10 tumors) per group). (PDF) [file pone.0123721.s005.pdf]

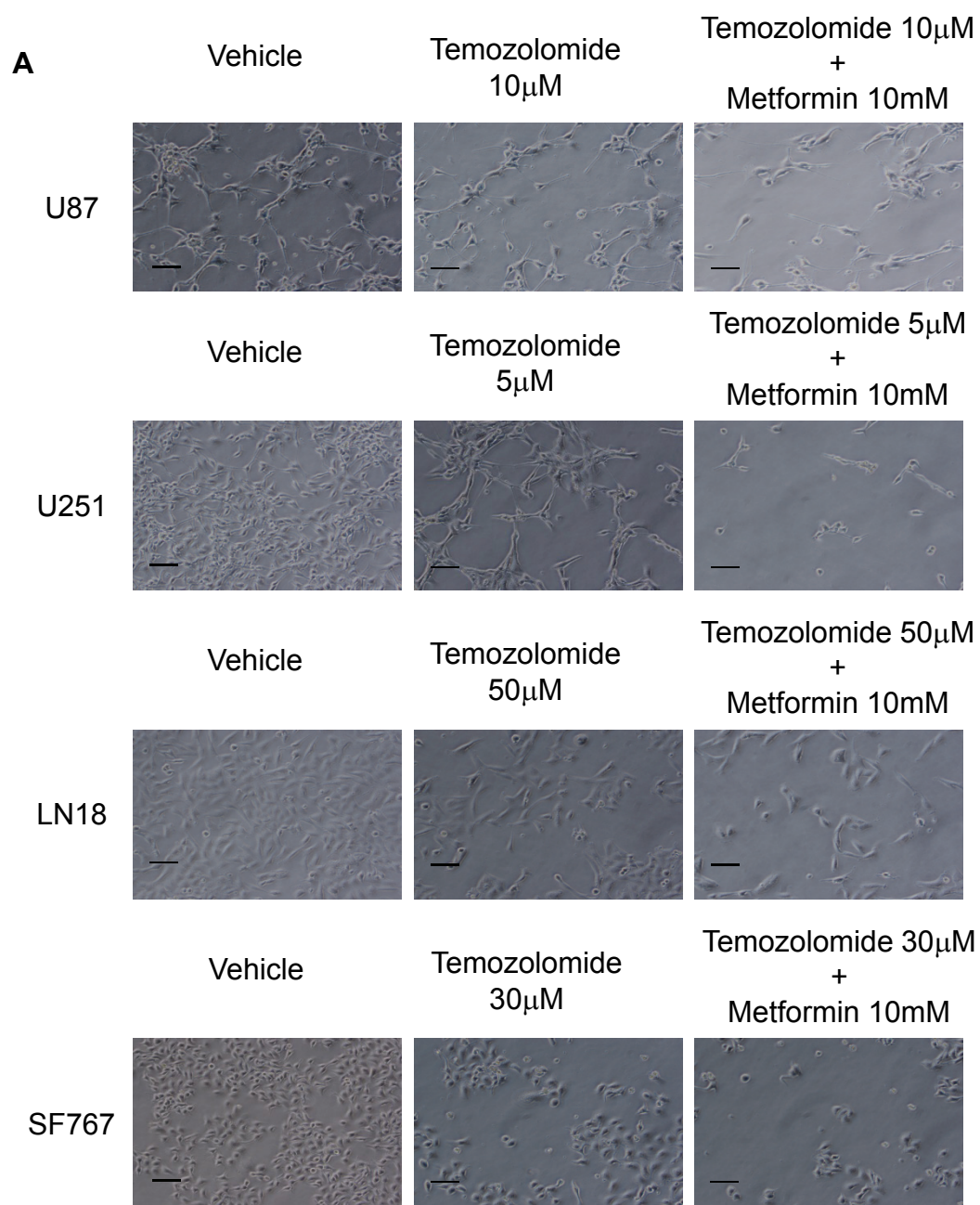

Supplement: S6 Fig — (A) Representative photographs of U87, U251, LN18 and SF767 cells treated or not with temozolomide (respectively, 10μM, 5μm, 50μm and 30μm) and/or metformin (10mM). Photographs were taken 48hrs after treatment (scale bars: 40μm). (PDF) [file pone.0123721.s006.pdf]

**A**

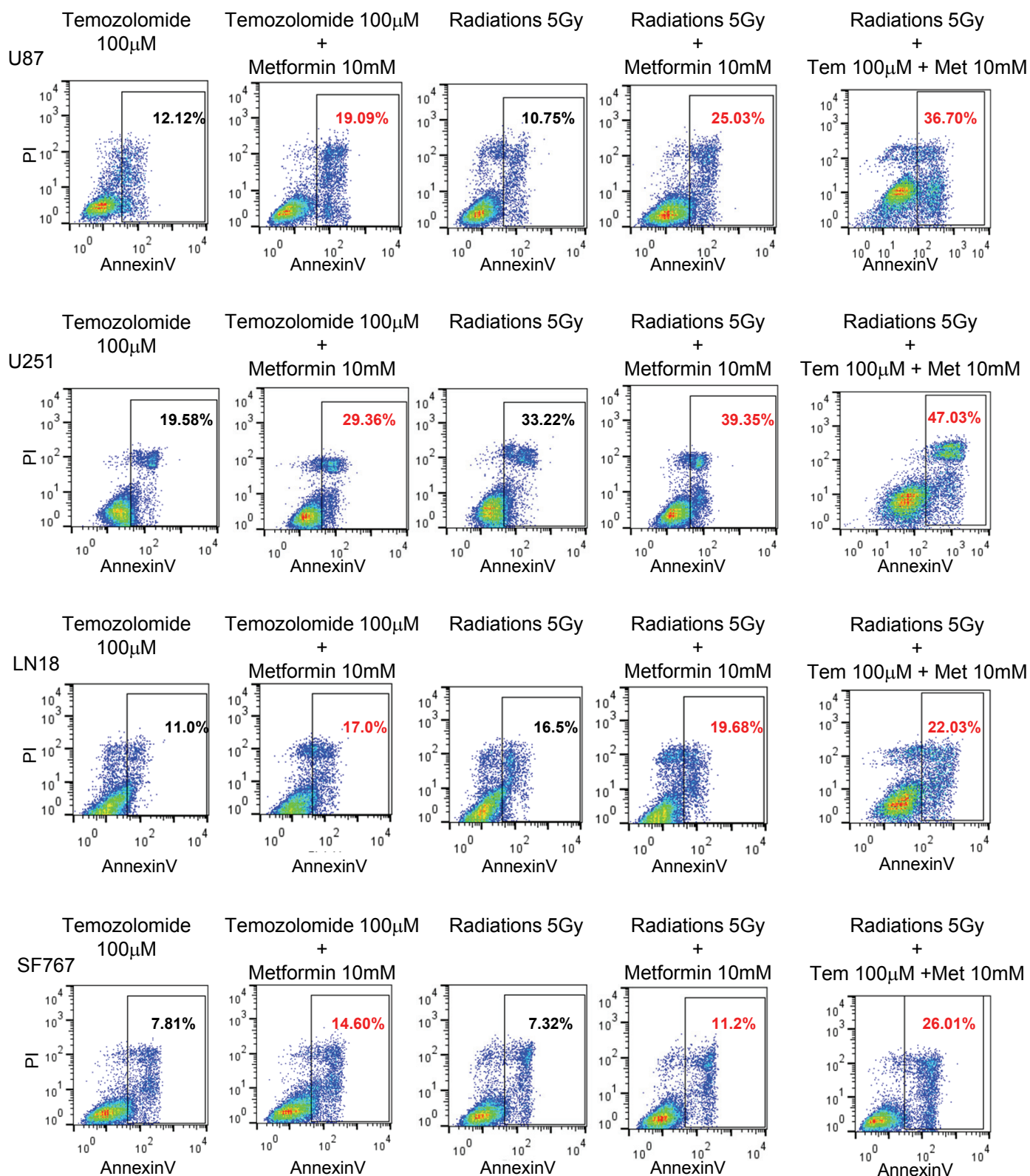

Supplement: S7 Fig — (A) Representative plots of Annexin-V/PI stained U87, U251, LN18 and SF767 cells treated or not with temozolomide (TMZ, 100μm), and/or radiations (IR, 5Gy) and/or metformin (10mM) for 48hrs. TMZ and/or IR combined with metformin present a stronger effect on cell death than TMZ or IR alone, particularly in U87, U251 and SF767 cells. (PDF) [file pone.0123721.s007.pdf]
